# Exploring Chemical Space Using *Ab Initio* Hyperreactor Dynamics

**DOI:** 10.1021/acscentsci.3c01403

**Published:** 2024-01-31

**Authors:** Alexandra Stan-Bernhardt, Liubov Glinkina, Andreas Hulm, Christian Ochsenfeld

**Affiliations:** † Chair of Theoretical Chemistry, Department of Chemistry, University of Munich (LMU), Butenandtstrasse 5, D-81377 München, Germany; ‡ Max Planck Institute for Solid State Research, Heisenbergstrasse 1, D-70569 Stuttgart, Germany

## Abstract

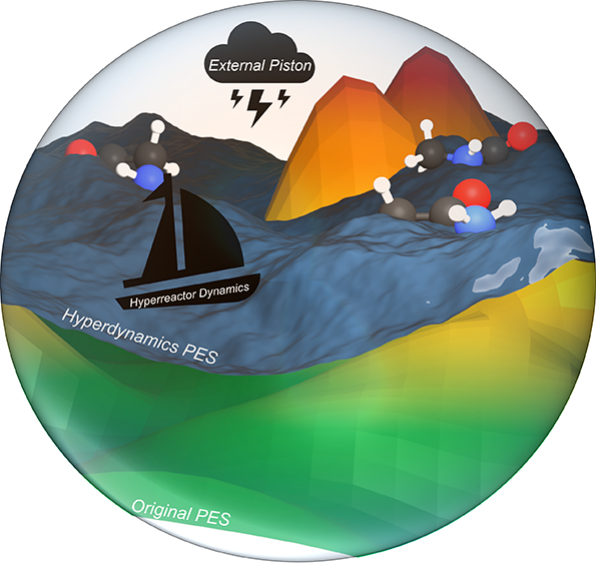

In recent years, first-principles exploration of chemical reaction space has provided valuable insights into intricate reaction networks. Here, we introduce *ab initio* hyperreactor dynamics, which enables rapid screening of the accessible chemical space from a given set of initial molecular species, predicting new synthetic routes that can potentially guide subsequent experimental studies. For this purpose, different hyperdynamics derived bias potentials are applied along with pressure-inducing spherical confinement of the molecular system in *ab initio* molecular dynamics simulations to efficiently enhance reactivity under mild conditions. To showcase the advantages and flexibility of the hyperreactor approach, we present a systematic study of the method’s parameters on a HCN toy model and apply it to a recently introduced experimental model for the prebiotic formation of glycinal and acetamide in interstellar ices, which yields results in line with experimental findings. In addition, we show how the developed framework enables the study of complicated transitions like the first step of a nonenzymatic DNA nucleoside synthesis in an aqueous environment, where the molecular fragmentation problem of earlier nanoreactor approaches is avoided.

## Abstract 2

We introduce hyperreactor dynamics to computationally navigate the vast chemical space of molecular systems on an elevated potential energy surface complemented by a pressure-inducing piston.

## Introduction

While theoretical validation of experimental results has become indispensable, breakthroughs in chemistry are still often achieved by exhaustive experiments based on trial-and-error, which are both time- and resource-consuming. Lately, predictive approaches have been emerging as powerful acceleration tools in biology, (bio)chemistry, physics, and materials science, e.g., to drive drug discovery and catalyst design.^1,2^ In particular, complex fields with many competing hypotheses, like the origins of life under prebiotic conditions,^3^ might profit from theoretical prediction. How, when, and where did life originate and which prebiotic molecular species played the key roles to allow for the emergence of life’s building blocks: amino acids and peptides, ribose, (oligo)nucleotides, and fatty acids? Valuable insights into these questions have already been gained by combining theoretical and experimental approaches, for instance by computationally reproducing the famous Miller–Urey experiment,^4−6^ investigating ribose formation within the formose network,^7,8^ or studying noncatalytic oligomerization of ribonucleotides.^9^

Therefore, exploiting the predictive power of computational chemistry by exploring the intricate chemical reaction space of a given set of compounds is a captivating endeavor. However, computational modeling of chemical systems is also highly challenging due to the high spatial dimensionality and almost infinite possibilities for reaction channels and possible conformations. Improvements in both software and hardware, as well as in available algorithms have helped the field evolve to a fast-paced study area underpinned by several recent developments in the available methodology and scope of application.^10^

The common principle of all first-principles chemical reaction space exploration methods is very simple: exhaustively investigate the mechanistic paths available to a set of initial compounds by simulating the system’s dynamics on its potential energy surface (PES) and construct the corresponding reaction network connecting all found intermediates and products by adjacent reaction paths.^11^ While first attempts to explore the vastness of the chemical reaction space by computational means relied on reducing the multidimensional problem of chemical transformations to a two-dimensional matrix representation paired with heuristic concepts,^12,13^ recent methodology^14−27^ leverages the power of available data science approaches, such as machine learning and neural networks,^28−30^ as well as efficiently exploits modern computer hardware.^31−37^

Out of all available chemical reaction space exploration approaches, we will focus on a molecular dynamics (MD) based quantum-chemical first-principles method, the *ab initio* nanoreactor,^6,19,38^ first introduced by Wang et al., which uses a temperature-based approach. Herein, reactivity is enhanced by performing high-temperature simulations of a spherically confined molecular system. In addition to employing temperatures of up to a few thousand Kelvin, an external virtual piston ensures the periodical contraction of the nanoreactor sphere, which in turn increases pressure and enables bond cleavage and formation as investigated by Wolinski and Baker.^39,40^ As the exploration proceeds undirected, the *ab initio* nanoreactor is a forward open-end exploration method according to the categorization presented by Unsleber and Reiher.^11^ However, only small molecular species can be studied as larger molecules can easily fragment under the extreme conditions employed.^8^

Interestingly, a similar sampling challenge is posed in the biochemical context. Here, to accelerate the simulation of slow processes, such as protein folding, numerous so-called enhanced sampling techniques have been developed.^41^ These rely either on elevating the PES along a predefined reaction coordinate to decrease reaction barriers locally or modifying the canonical probability distribution of the whole system, eliminating the need for prior knowledge about the involved collective variables (CVs). The latter strategy is used in temperature-based methods like replica exchange^42^/parallel tempering^43^ (RE/PT) MD^44^ or integrated tempering sampling (ITS),^45,46^ as well as in bias potential-based methods, e.g., hyperdynamics,^47,48^ accelerated MD^49^ (aMD) and its successors, Gaussian accelerated MD^50^ (GaMD), and Sigmoid accelerated MD^51^ (SaMD). The hyperdynamics scheme by Voter et al.^47,48^ is based on identifying transition regions and elevating them by a boost potential dependent on the Hessian matrix and approximations thereof. Hamelberg et al.^49^ provides in aMD a different form for the bias potential to increase computational efficiency. Later, more general formulations for the bias potential were presented in GaMD^50^ and SaMD^51^ with the goal to improve sampling efficiency and accuracy.

Enhanced sampling methods using bias potentials along a predefined CV have already been successfully employed for automated chemical reaction space exploration as demonstrated by Grimme in his metadynamics-based approach, where the RMSD was employed as a CV.^52^ Herewith, valuable insights into the catalytic behavior of a P450 induced oxidation were achieved. Furthermore, Hirai and Jinnouchi used an adaptive form^53^ of the aMD method by Hamelberg et al.^49^ in combination with increased temperatures within the previously described nanoreactor framework to study the oxidation-induced decomposition of ethylene at 1273 K.^54^ They also extended their method to the treatment of solid state reactions,^55^ highlighting the potential of enhanced sampling methods to aid modeling.

Our aim is to extend our previous investigations on the *ab initio* nanoreactor approach, which involved the development of a new, milder form for the virtual piston, along with an automated simulation postprocessing workflow to highly speed up the generation of reaction networks from nanoreactor trajectories.^8^ For this purpose, we combine spherical confinement with hyperdynamics-inspired biasing strategies in an approach which we refer to as *ab initio* hyperreactor dynamics (HRD). This provides a robust method for conducting reaction space exploration under mild conditions.

## Theoretical Background and Methods

### Reaction Space Exploration With the Computational Nanoreactor

Based on the original nanoreactor developed by Martínez and co-workers,^6^ we have presented a systematic parameter study^8^ which also includes a comparison of different forms for the potential controlling the aforementioned virtual piston to assess its effect on the obtained reactivity and numerical stability of the simulation. In this context, we have introduced a new, milder form of the contracting spherical potential *V*^sphere^
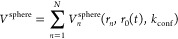
1where *N* denotes the number of atoms. The magnitude of *V*_*n*_^sphere^ acting on each atom *n* depends on the atomic radial coordinate *r*_*n*_, as well as on the imposed time-dependent radius for the nanoreactor sphere *r*_0_(*t*), to which the atoms are confined.
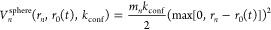
2
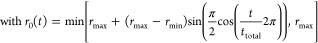
3To ensure equal acceleration of all atoms at the same radial coordinate *r*_*n*_, the applied harmonic potential *V*_*n*_^sphere^ is additionally mass-weighted by a unitless factor equal to the magnitude in atomic units (a.u.) of the corresponding atomic mass *m*_*n*_. The radius of confinement *r*_0_(*t*) oscillates according to eq 3 between *r*_min_ and *r*_max_, following a smooth-step curve with a period of *t*_total_.^8^

This form for the temporal switch of the external potential and the corresponding spherical confinement has enabled increased stability of the performed simulations^8^ compared to the rectangular wave potential used in the original nanoreactor procedure.^6^ Therefore, the former was employed in the present study to induce pressure at low thermostat temperatures *T*_target_ along with the flattening of the potential energy surface by hyperdynamics-inspired methods^49−51^ in HRD, as presented in the following.

### Enhanced Sampling by Hyperdynamics-Related Methods

The hyperdynamics method of Voter^47,48^ mentioned before was deemed unfeasible for enhancing the sampling of large systems due to the related increased computational cost. This led to the proposal of a trivial condition for applying a boost potential to lift the PES in minimum energy wells by Steiner et al.^56^ and its application to surface diffusion processes. Rahman and Tully^57^ showcased the effectivity of the latter method which they termed “puddle-skimming” for exploring the multidimensional configuration space by adding a boost potential Δ*V*(**x**) to the original potential *V*(**x**) (where **x**^T^ = (*x*_1_, *x*_2_, *x*_3_, ..., *x*_3*N*_)) above a certain threshold value:
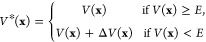
4Herein, Δ*V*(**x**) = *E* – *V*(**x**) and *E* is the boost energy, below which the bias is applied to aid the escape from minimum energy wells. Therefore, the PES forms a “puddle” if *V*(**x**) < *E* where the molecular system experiences a random walk, as V*(**x**) = *E*. However, this simple definition for Δ*V*(**x**) leads to discontinuities in the first derivative of the modified potential at the points where *V*(**x**) = *E* and, therefore, requires a special integration technique to ensure temporal propagation on the PES during the MD.

Since then, several formulations for Δ*V*(**x**), which do not exhibit the above-mentioned discontinuities, have been proposed and applied to enhance the conformational sampling of biomolecules and enable the investigation of slow processes on computationally feasible time scales. Herein, we will focus on the aMD method^49^ and its successors, GaMD by Miao et al.,^50^ and the recent SaMD approach presented by Zhao et al.^51^

The formulations for Δ*V*(**x**) as introduced in eq 4 and its derivative with respect to *V*(**x**) for the three enhanced sampling methods mentioned above are summarized in Table 1. To ensure proper sampling during the simulation, the added potential should modify the PES in such a way that the underlying shape is preserved to a certain degree. The parameter *E* should be defined carefully, as if chosen below *V*_min_ (the local minimum near the starting structure) the modified potential will always remain unbiased.

**Table 1:** Formulations of the Boost Potential Δ*V* and Its Derivative with Respect to *V* for aMD,^49^ GaMD,^50^ and SaMD^51^

method	Δ*V*(**x**)	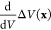
aMD^49^	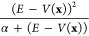	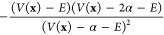
GaMD^50^	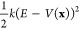	–*k*(*E* – *V*(**x**))
SaMD^51^	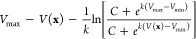	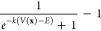

In aMD, the parameter α defines the strength of the added bias, so that the modified potential transitions from a flat to the true PES for increasing values of α. Even though aMD provides a faster and more comprehensive method to explore the conformational and reaction space of large systems compared to conventional MD simulations, in many cases accurate free energy differences of the simulated processes are desired. To obtain the latter, the previously added bias must be removed. Unfortunately, due to the large boost potentials applied in aMD, which range between tens and hundreds of kilocalories per mole and also possess a wide distribution, this reweighting step has proven to be problematic and suffer from large energetic noise,^58^ which makes recovering the accurate free energy landscapes challenging.^49^

Therefore, GaMD aims to provide improved reweighting by using a harmonic boost potential Δ*V*(**x**) which follows near-Gaussian distribution.^50^ Finally, the recently introduced SaMD approach targets the improvement of sampling efficiency and effectivity of the applied boost potential while minimizing its standard deviation. As its name suggests, a sigmoid form was chosen for the first derivative of the boost potential (compare Table 1 third column, third row) with respect to *V*(**x**). For both GaMD and SaMD, the strength of the boost potential, and therefore of the applied bias, is controlled by a user-defined standard deviation σ_0_ for Δ*V*(**x**), which in turn determines *k* as described in the Supporting Information.

**Figure 1:**
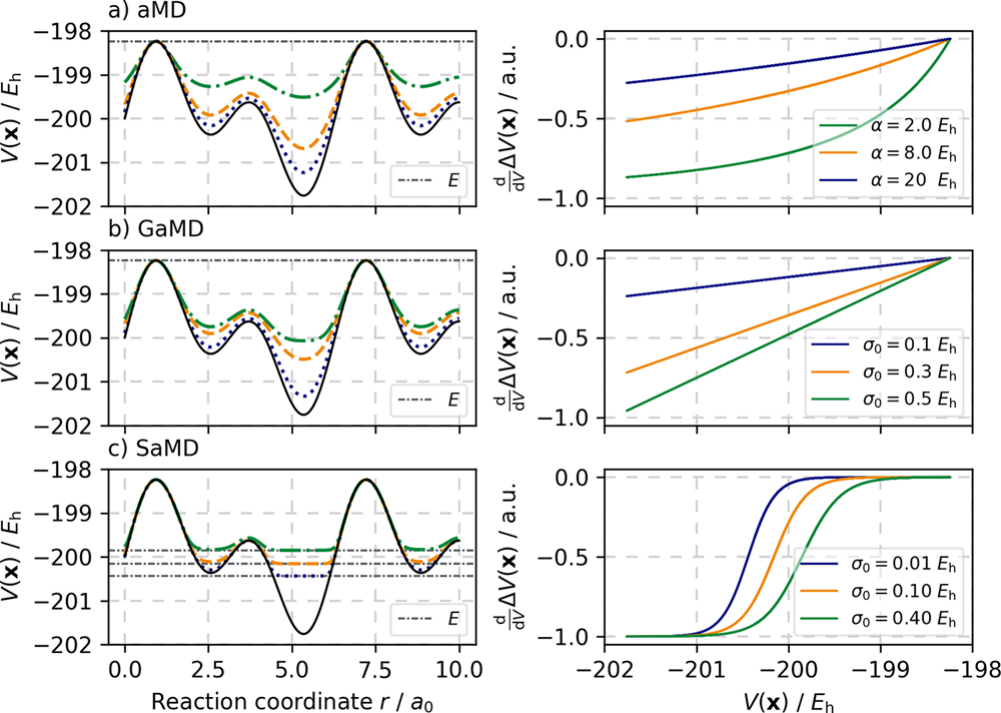
Boost potential applied to a hypothetical potential and derivative with respect to *V*(**x**) of corresponding bias forces for aMD (top), GaMD (middle), and SaMD (bottom).

For better understanding the three hyperdynamics-inspired techniques, the different boost potentials Δ*V*(**x**) are plotted for a hypothetical potential on the left of Figure 1, along with the derivative with respect to *V*(**x**) of the corresponding bias force on the right. For all presented approaches, the boost energy *E*, along with the minimal and maximal potential energy, *V*_min_ and *V*_max_, are estimated from the calculated potential during the equilibration simulation performed in the beginning.

### Inducing Reactivity with Accelerated MD Methods

While both the computational nanoreactor method as introduced by Wang et al.^6^ and the adaptive aMD approach used by Hirai^53^ provide good results for small reactants and have proven to be of great use for simulating combustion, the extreme temperatures of thousands of Kelvin employed therein, which aid the reactivity by increasing the kinetic energy of the system, also unavoidably increase the risk for numerical instability and molecular fragmentation.

Our aim is to achieve similar reactive behavior as in the high-temperature approaches while keeping the target temperature of the thermostat low and transit to a more general reaction space exploration method. Hyperdynamics-related methods, such as aMD, GaMD, and SaMD provide useful tools for inducing reactive behavior without the need of choosing reaction coordinates *a priori*.

**Figure 2:**
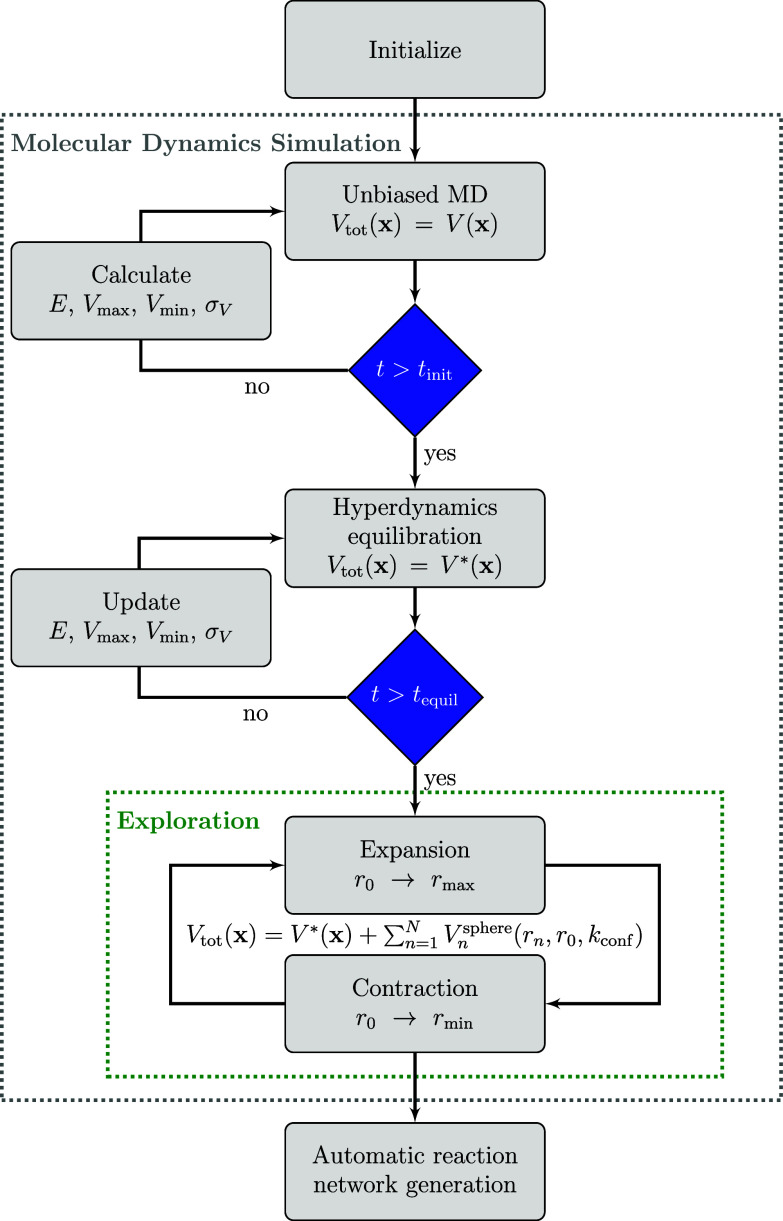
Flowchart of the HRD procedure consisting of applying a periodic contraction on the molecules confined to a virtual nanoreactor sphere after having already biased the original potential using aMD, GaMD, or SaMD boost potentials. While for *t* ≤ *t*_init_ the original PES is preserved, the two bias potentials complement each other during the exploration phase after the equilibration of the chosen hyperdynamics procedure has been finished (*t* > *t*_eq_). The boost potential Δ*V*(**x**) is applied in each MD step prior to the external harmonic potential *V*^sphere^.

Figure 2 gives a quick and comprehensive overview of the workflow employed in accelerated HRD (aHRD), Gaussian accelerated HRD (GaHRD), and Sigmoid accelerated HRD (SaHRD) depending on the chosen boost potential Δ*V*(**x**). The definitions for V*(**x**) and *V*_*n*_^sphere^(*r*_*n*_, *r*_0_(*t*), *k*_conf_) are given in eq 2, eq 4, and Table 1. We expect this to enable investigation of a broader range of systems and extend the scope of the present temperature-based computational nanoreactor approach.

For the investigated systems, only selected products, intermediates, and reaction paths are included in the results. The focus of the present work has been on the exploration of chemical space and the comparison of the induced reactivity by the different hyperdynamics-inspired bias potentials. Therefore, our goal for the future is to enable (semi)automated refinement of the obtained reaction networks and paths of interests as already introduced by Martínez and co-workers^59,60^ for the conventional nanoreactor approach. For this purpose, we aim to leverage the ongoing efforts in our group to reduce computational scaling of accurate quantum chemical calculations, e.g., at the DFT level,^31−33^ to accelerate the refinement procedure.

## Computational Details

All simulations included in this study were performed using the GFN2-xTB method^61^ for the computation of energies and gradients, whereby xtb(62) was interfaced to our in-house MD engine. Default settings were used for the SCF convergence criterion (Δ*E*_SCF_ ≤ 10^–6^*E*_h_) and for the electronic temperature *T*_el_ = 300 K. The initialization in the nanoreactor sphere was done using preoptimized molecules at the PBEh-3c^63^/def2-mSVP level of theory using our initialization procedure introduced before.^8^

The implementation of aMD, GaMD, and SaMD boost potentials and forces was provided by the adaptive-sampling package,^64,65^ where the implementation was done as described in the original publications.^49−51^ The postprocessing of the simulations was fully automated by using our nanoreactor evaluation workflow.^8,66^

### Simulation Details

For the method comparison of aHRD, GaHRD, and SaHRD, we have conducted triplicates for all tested parameters. For this purpose, a homogeneous test system of 50 HCN molecules enclosed in a theoretical nanoreactor sphere contracting between 15 and 7 Å was used and a Langevin thermostat at *T*_target_ = 298.15 K with a friction constant of γ = 7 ps^–1^ was employed for temperature control. For all simulations, 1 × 10^3^ initial steps were used, while the number of equilibration steps was set to 1 × 10^4^. The external spherical confinement was turned on after the equilibration was finished and σ_*V*_, *E*, *V*_min_, and *V*_max_ had been determined. The force constant *k* was computed for GaHRD and SaHRD as described in the Supporting Information. The interstellar low-temperature system consisting of acetaldehyde, ammonia, and radical counterparts was simulated at the reported^67^ experimental temperature of 10 K in vacuum. For the simulation of the nonenzymatic nucleoside synthesis, GaHRD and SaHRD simulations in vacuum and using implicit water solvation (ALPB model)^168^ at *T*_target_ = 323.15 K were compared to conventional nanoreactor simulations at *T*_target_ = 2000 K. Full details on all simulation parameters, as well as the initial geometries used for the HCN system, are given in the Supporting Information.

## Results and Discussion

The newly developed HRD method is first tested extensively at *T*_target_ = 298.15 K on the literature-known^8^ HCN molecular setup consisting of 50 molecules (150 atoms), and the outcome is compared to the results obtained with the high-temperature *ab initio* nanoreactor procedure. Furthermore, to ensure good temperature and pressure control, we investigate the behavior of the HRD procedure at different equilibrium temperatures. The efficiency of HRD simulations is then showcased on two prebiotically relevant application setups, concerning the synthesis of indispensable molecular building blocks for the emergence of life.^3^

### Exploring Reactivity Enhancement

To investigate the required strength for driving reaction space exploration by combining aMD, GaMD, or SaMD boost potentials with the periodic contracting external potential introduced in the context of the computational nanoreactor,^6,8^ a set of nine suitable acceleration parameters was determined for each HRD type by running unbiased MD simulations of the HCN test system described above and visualizing V*(**x**) against the unbiased potential energy surface. The aim was to test a wide range of acceleration parameters in terms of quantitative and qualitative reactivity outcome.

The chosen maximal trajectory length for the HRD exploration phase was 100 ps, including the equilibration period of 5 ps. Due to the varying trajectory lengths caused by different bias strength and resulting numerical problems, we report the number of new unique molecular species obtained every 2 ps, which corresponds to the period of the contracting spherical potential *V*^sphere^. By applying this criterion, we aim to partially remove distortions caused by different simulation lengths.

**Figure 3:**
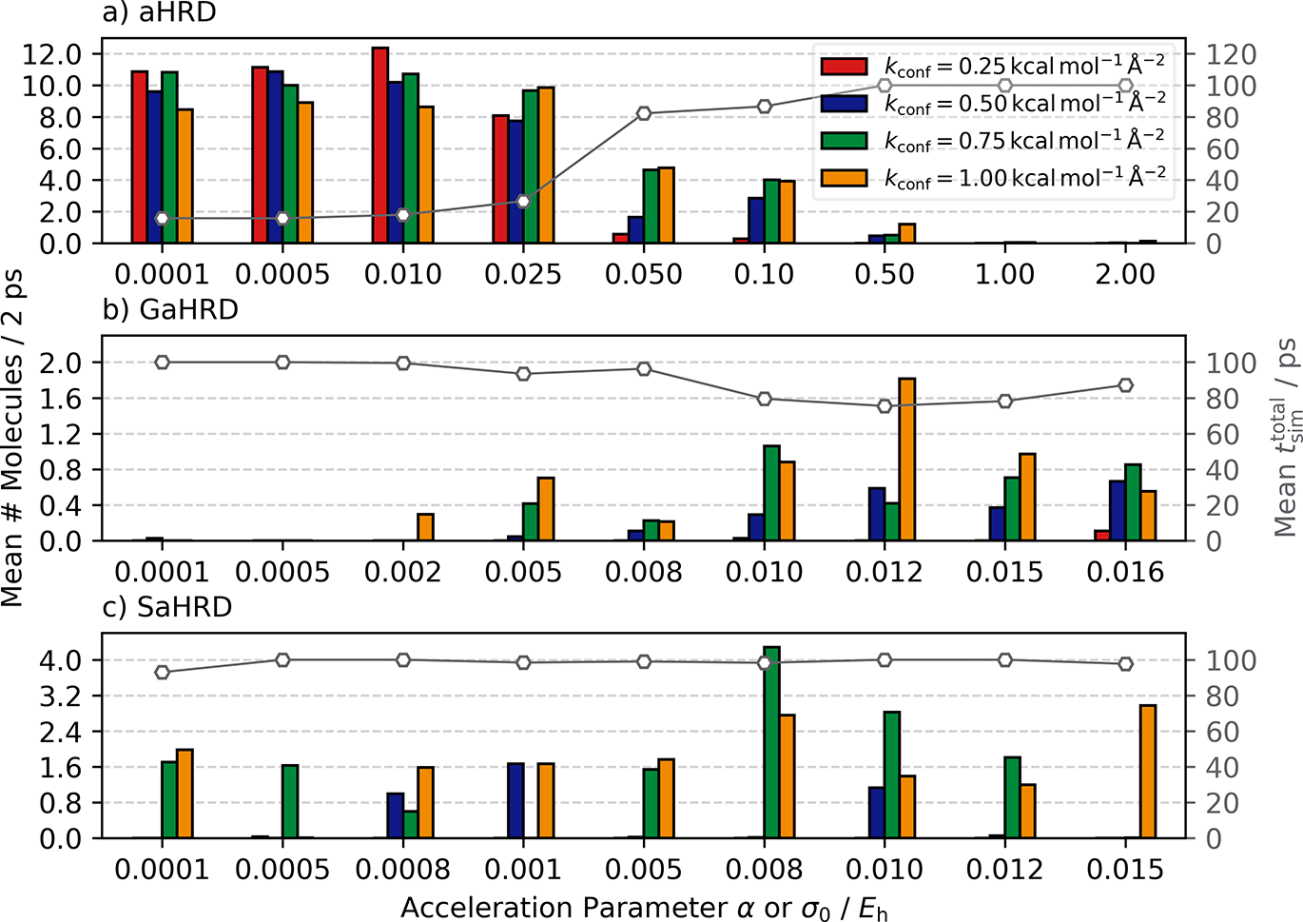
Results reported as number of new unique molecular species obtained every contraction–expansion period (2 ps) for HRD simulations employing different confinement force constants *k*_conf_ and the nine selected acceleration parameters for each of the different HRD variants. The total simulation time was averaged for each acceleration parameter over four simulations employing different *k*_conf_. Low values for α induce great reactivity; however, they also lead to instability of the simulations and total simulation times of under 50 ps as highlighted in dark gray. The Gaussian accelerated approach provides the best balance between acceleration and stability by showing low sensitivity to the choice of *k*_conf_, while SaHRD delivers the best qualitative outcome overall (see Figure 4).

The results obtained for aHRD, GaHRD, and SaHRD simulations of the test HCN system at 298.15 K and *k*_conf_ = 1.00 kcal mol^–1^ Å^–2^ are shown in Figure 3 in orange. The achieved total simulation time is shown in dark gray for better interpretation of the results. An additional figure providing an enlargement for this confinement strength is given in Figure S2. We note that due to the computational effort needed to perform these simulations, we have restricted the number of parallel simulations to three per setup. Even though the results are not statistically converged and outliers were encountered, the performed simulations provide useful trends. The reactivity enhancement correlates as expected with the employed acceleration parameter α for aHRD and σ_0_ for GaHRD/SaHRD, as the number of obtained molecular species increases for decreasing α and increasing σ_0_, respectively. Furthermore, both reactivity and stability are clearly more sensitive to α than to σ_0_, which represents a user-defined upper threshold for the maximal standard deviation of the boost potential Δ*V*(**x**). Low values for α highly increase the reactivity; however, low stability of the simulation is observed, which leads to numerical problems. As expected from the behavior of , SaHRD exhibits the lowest sensitivity to the chosen σ_0_ by allowing a random walk in the flattened basins of the PES (compare Figure 1 c and Table 1).

As we employ two reactivity-enhancement techniques for the HRD method, we have additionally performed calculations to show the interplay of the spherical confinement and the chosen strength of the hyper-MD bias. For this purpose, we compare the previously obtained results at *k*_conf_ = 1.00 kcal mol^–1^ Å^–2^ to the outcome when gradually reducing the confinement strength to *k*_conf_ = 0.25 kcal mol^–1^ Å^–2^. The results confirm earlier findings, that increasing the confinement strength in general aids the exploration process.^8^ However, it can also hinder it in the case of HRD when the two bias potentials become too strong together, leading to increased numerical problems and early termination of the simulations, or they cancel each other, therefore, hindering the exploration of new regions as shown in Figure 3. Considering these findings, we recommend using the highest confinement possible while applying a medium-strength acceleration parameter. This should provide a good balance between numerical stability of the simulation and induced reactivity.

**Figure 4:**
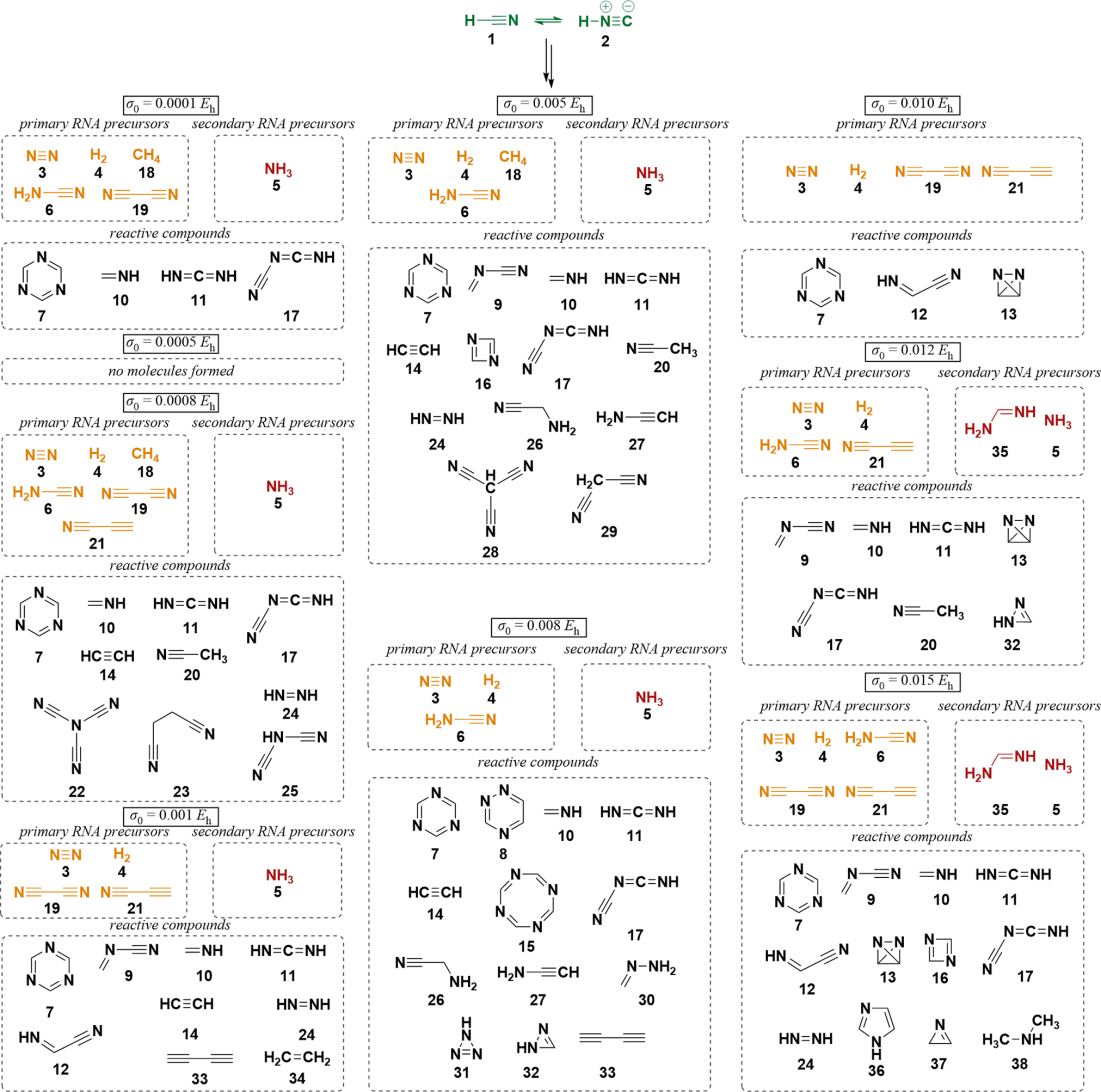
Selection of obtained molecular species for SaHRD simulations at *k*_conf_ = 1.00 kcal mol^–1^ Å^–2^ and *r*_min_ = 7 Å/*r*_max_ = 15 Å, ranging from simple carbohydrates to RNA precursors as postulated by Benner et al.,^68^ and important heterocyclic compounds, such as imidazole (**36**). Starting compounds are shown in green, while primary and secondary RNA precursors are depicted in orange and red, respectively.

From the three different tested HRD approaches, SaHRD performed best both quantitatively for the ideal *k*_conf_ = 1.00 kcal mol^–1^ Å^–2^, as shown in Figure 3 in orange, and qualitatively by providing a wide range of compounds, including previously identified RNA precursors with the earlier piston-accelerated nanoreactor approach^8^ at 2000 K; e.g., methane (**18**), ethanedinitrile (**19**), formamidine (**35**), and cyanoacetylene (**21**) were found along with imidazole (**36**) when increasing the bias strength. A selection of identified compounds is summarized in Figure 4 based on the classification in primary and secondary RNA precursors presented by Benner et al.^68^ Selections for compounds obtained within aHRD and GaHRD simulations can be found in the Supporting Information, where an overview of numbered molecules obtained in all conducted HCN simulations within this work is also provided.

### Temperature and Pressure Regulation in Hyperreactor Dynamics Simulations

To check temperature and pressure regulation by the employed Langevin thermostat (*T*_target_ = 298.15 K; γ = 7 ps^–1^), we looked at the mean temperature and pressure throughout the simulation for every combination of *k*_conf_ and α/σ_0_. Outliers were removed prior to the analysis by the IQR method.^69^ The results shown in Figure S3 reveal very good temperature control for most simulation setups except for two aHRD and two SaHRD cases, which are clearly outliers and caused by the very harsh bias employed. Furthermore, the pressure fluctuations have a clear tendency to increase for higher *k*_conf_, as expected and reported before,^8^ as well as for milder acceleration during the hyperdynamics. This finding can be attributed to increasing spherical confinement due to atom dissipation, and it systematically shows the interplay between the two bias potentials.

**Figure 5:**
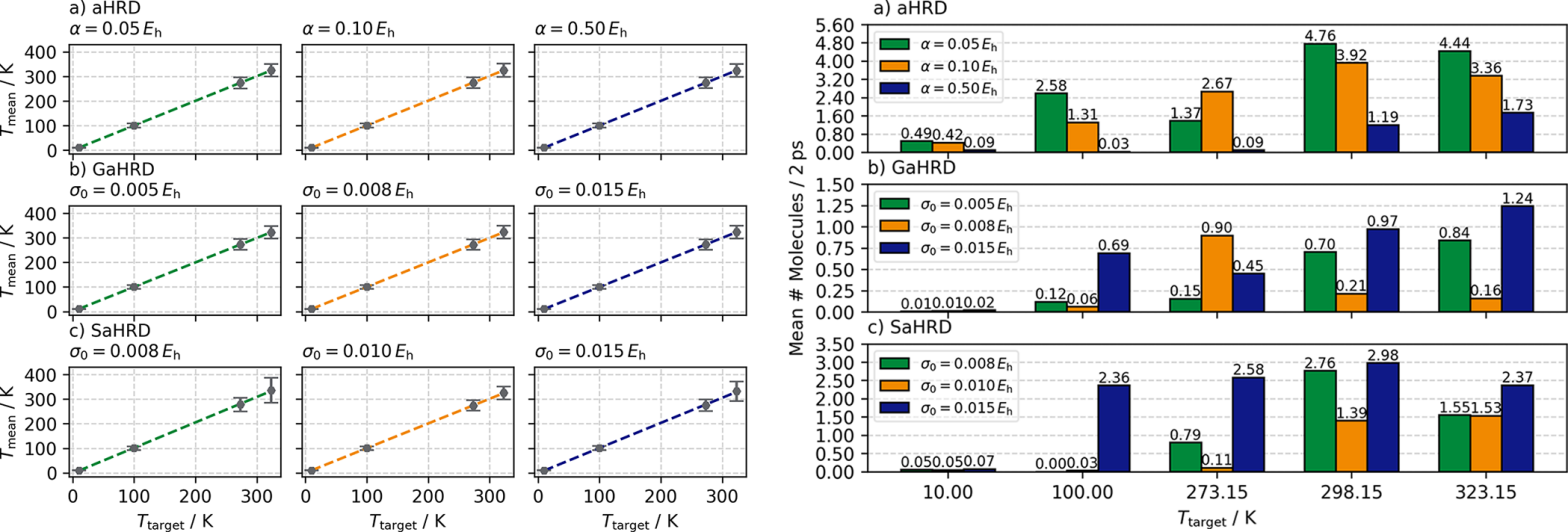
Investigations on HRD reaction space exploration at different potentially experimentally defined equilibrium temperatures. Excellent temperature regulation is achieved by the Langevin thermostat for very low temperatures down to 10 K. Reactivity is enhanced, as expected, by higher temperatures and acceleration strength. All simulations were conducted with *k*_conf_ = 1.00 kcal mol^–1^ Å^–2^ and *r*_min_ = 7 Å/*r*_max_ = 15 Å.

To check the performance of the HRD approach under varying equilibrium conditions, we have conducted investigations at four different temperatures, *T*_target_ = 10.00, 100.00, 273.15, and 323.15 K using the best performing setups from previous findings. Notably, the Langevin thermostat precisely regulates the temperature regardless of the employed acceleration parameter as shown on the left of Figure 5. This provides an excellent basis for conducting low temperature reaction space exploration studies.

In terms of obtained results, there is no clear correlation between the number of obtained unique molecular species every 2 ps and the employed equilibrium temperature paired with previously defined acceleration parameters for either aHRD, GaHRD, or SaHRD simulations. The reactivity tremendously increases regardless of the employed acceleration strength, when surpassing *T* = 100.00 K. We attribute this to the used test system, and it should not represent a general rule. Even though SaHRD shows low sensitivity to the choice of σ_0_ at *T* = 298.15 K in terms of simulation stability, the chosen temperature induces numerical problems when exceeding 300 K for the chosen test setup. Nevertheless, the obtained results were very satisfactory for all three methods and show that choosing the right temperature when aiming to observe certain chemical transformations using the HRD approach is crucial.

In addition, we have investigated the computational feasibility of aHRD, GaHRD, and SaHRD for the three best-performing acceleration parameters presented above compared to conventional nanoreactor simulations of the HCN system at 298.15 and 2000.00 K. The HRD simulations were conducted at an equilibrium temperature of 298.15 K. The results presented in Figure S1 reflect our previous observations regarding stability and reactivity of the simulations. The increased target temperature in the conventional nanoreactor approach leads to a computational overhead due to additional steps needed to heat and equilibrate the system prior to conducting the exploration phase, as well as plays a significant role in destabilizing the simulation and leading to numerical problems, therefore, also increasing the computation time. Furthermore, even though the type of the bias potential does not influence the performance, the suitability of the chosen acceleration parameter clearly does and the choice of the latter should therefore be made with care. On this note, we conclude that HRD delivers better performance for a wider variety of chemical systems than the conventional nanoreactor approach, as it allows for efficient reaction space exploration at low temperatures, therefore providing better numerical stability of the simulation and solving the problem of molecular fragmentation.

### Application to Origins of Life Examples

To showcase the efficiency of HRD, two experimental setups were investigated at the experimental equilibrium temperatures.^67,70^ While the first system was chosen due to its extreme low temperature conditions, the second aimed to simulate reactions involving combined implicit-explicit solvation, more complex reactants prone to severe fragmentation, and a moderate experimental temperature of 50 °C.

#### Prebiotic Synthesis of Glycinal and Acetamide in Interstellar Ices

**Figure 6:**
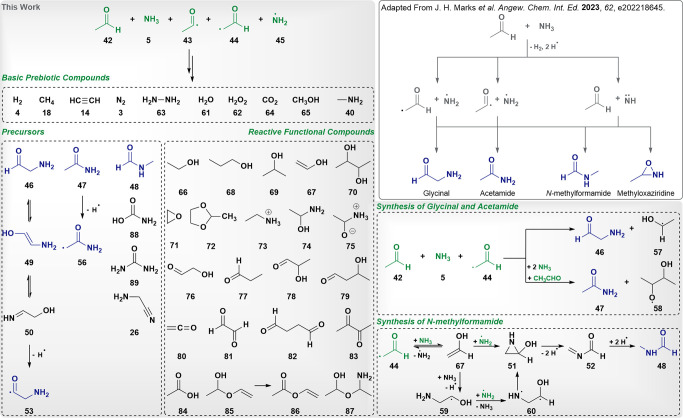
Comparison of obtained molecular species and selected reactions in aHRD, GaHRD, and SaHRD simulations at *T* = 10 K (gray background) and experimentally obtained products on the upper right. The simulations were started from varying mixtures of acetaldehyde, ammonia, and radical counterparts shown in green. Obtained peptide precursors matching experimental observations^67^ are depicted in dark blue.

In a recent study,^67^ R. I. Kaiser and co-workers presented a novel possible interstellar access pathway for glycinal and acetamide initiated by simulated galactic cosmic rays in interstellar model ices at 10 K. These two molecular species represent important molecular building blocks for the formation of glycine units in prebiotic polymeric chains. The experimentally established procedure supports radical formation by irradiation of a mixture of acetaldehyde and ammonia leading to radical species by hydrogen elimination and subsequent recombination of the obtained radicals to glycinal, acetamide, *N*-methylformamide, and methyloxaziridine as shown in Figure 6 (upper right). These intermediate products can undergo barrierless recombination followed by rearrangement, ultimately yielding amide chains.

Given our preliminary investigations on the behavior of HRD at low equilibrium temperatures, we have conducted a study using different setups for the interstellar formation of glycinal, acetamide, and amides starting from acetaldehyde (**42**), ammonia (**5**), as well as including corresponding radical species (**43**, **44**, **45**). For this purpose, aHRD, GaHRD, and SaHRD simulations were performed at an equilibrium temperature of 10 K using different acceleration parameters. While for aHRD, α was determined through unbiased test simulations, for GaHRD and SaHRD we have decided to compare σ_0_ values ranging between 10 *k*_B_*T*, which represents the recommended upper threshold for correct reweighting, and 100 *k*_B_*T*. All further details on simulation parameters are given in the Supporting Information.

All tested setups support the experimental data with glycinal (**46**), acetamide (**47**), as well as radical counterparts (**53**, **56**) being formed. New reaction paths potentially leading to glycinal and acetamide could be identified in all simulations regardless of the applied reactivity enhancement procedure. However, as already observed in the parameter screening using the homogeneous HCN setup, determining the right acceleration strength for aHRD is challenging and it highly relies on chemical intuition.

In addition to the two expected amide precursors mentioned before, 1-aminoethanol (**74**) and hemiacetals (**85**, **87**) were formed in the radical-free simulations starting from a mixture of acetaldehyde and ammonia in a ratio of 13:14 (147 atoms). Herein, ammonia was often seen to act as a catalytic species.

To investigate radical reactions in this context, simulations of two systems involving different reactant-derived single radical species mixed with acetaldehyde and ammonia were conducted. Ratios of 6:1 and 1:1 of nonradical to radical species (shown in Figure 6 in green) were tested. Here, various reaction paths ranging from barrierless radical recombinations to nucleophilic additions and rearrangements involving heterocyclic species (**51**) could be identified. A wide variety of prebiotic compounds, such as hydrogen (**4**), nitrogen (**3**), methane (**18**), acetylene (**14**), hydrazine (**63**), water (**61**), carbon dioxide (**64**), methanol (**65**), and methylamine (**40**), was retrieved from the 300 ps-long simulations regardless of the employed acceleration parameters, merely the time of formation differing. Besides the obtained postulated precursors, namely glycinal, acetamide, and *N*-methylformamide (**48**) shown in dark blue in the lower left corner in Figure 6, numerous other reactive molecular species exhibiting various functional groups, such as alcohol, amine, (hemi)acetal, and carbonyl groups were obtained. These reactive compounds, among which glyoxal (**81**), 1-aminoethanol (**74**), and glycolaldehyde (**76**) were present, play a crucial role in driving reactivity during the exploration phase by providing protons and nucleophilic character.

Furthermore, carbamic acid (**88**) and urea (**89**), as well as aminoacetonitrile (**26**), which is a precursor of glycine, could be identified in the SaHRD simulations and provide a good molecular starting pool for the prebiotic nonenzymatic formation of amino acid chains. Overall, the performed HRD simulations were very efficient, GaHRD performing the best in terms of convergence and stability of the simulations which can be attributed to the biased potential preserving the topology of the PES. Regarding molecular variety, SaHRD performed best by additionally supporting the formation of longer carbon chains besides the expected products, therefore leading to species such as butanedial (**82**) and vinylacetate (**86**). However, in the case of SaHRD, increasing instability was encountered for simulations performed with stronger bias. We attribute this behavior to the flat topology of the modified PES at *V*(**x**) = *E*, as well as to the strong bias of up to σ_0_ = 100 *k*_B_*T* employed.

Three selected reaction paths leading to glycinal, acetamide, and *N*-methylformamide are shown in the lower right corner of Figure 6. While the former two compounds were obtained from acetaldehyde, ammonia, and a formylmethyl radical through nucleophilic attack at the radical species followed by hydrogen transfer, the synthesis of the latter involved a cyclization to 2-aziridinol (**51**) which enabled the rearrangement to *N*-methyleneformamide (**52**), and ultimately to *N*-methylformamide (**48**).

#### Nonenzymatic Synthesis of DNA Nucleosides

**Figure 7:**
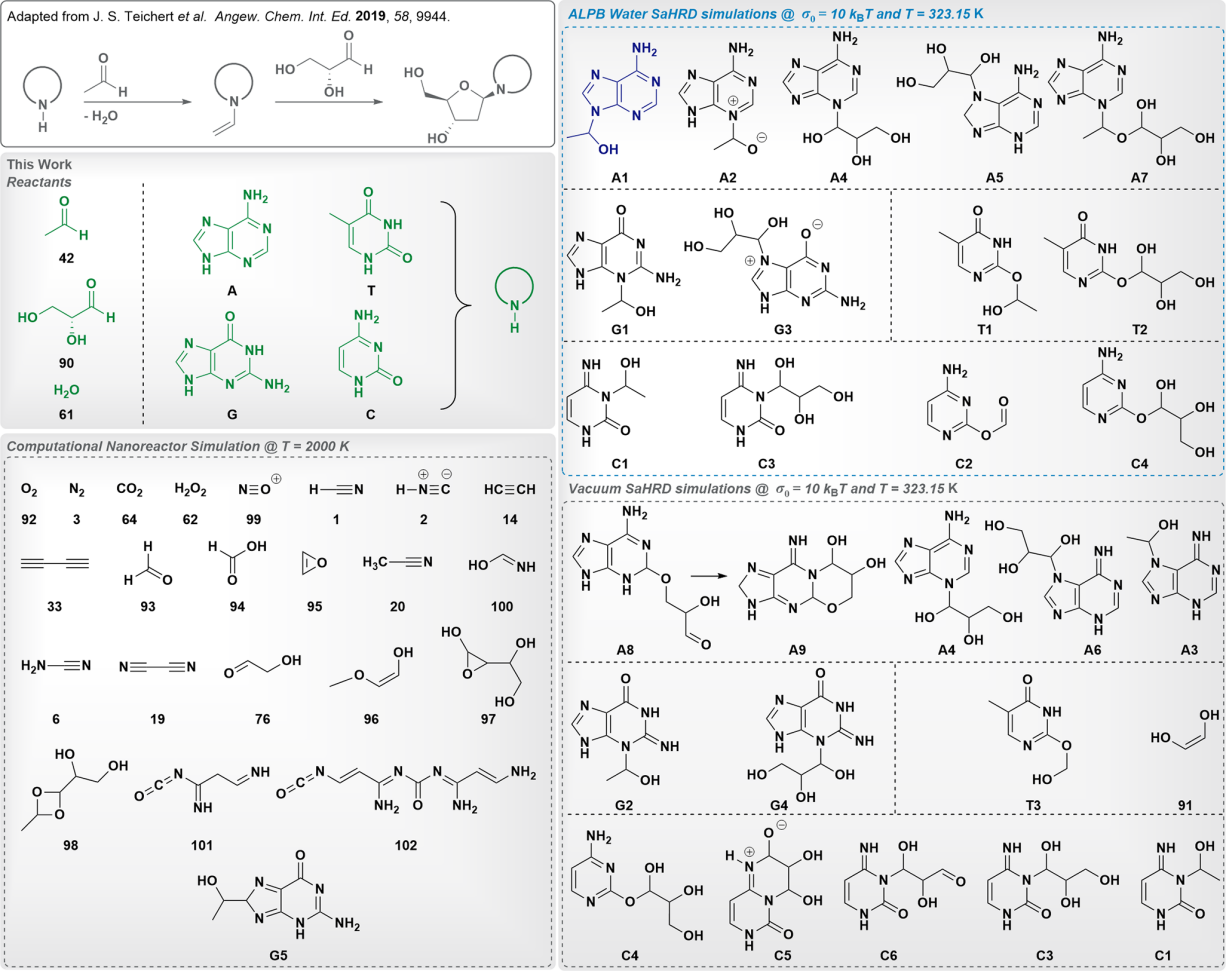
Results of SaHRD simulations investigating a nonenzymatic DNA nucleoside synthesis (experimental results shown in the upper left corner) at *T* = 323.15 K and using σ_0_ = 1.023 × 10^–2^*E*_h_ (corresponds to 10 *k*_B_*T*). Reactants are given in green. Simulations were performed with and without the ALPB implicit solvent model for water, which is shown to support nucleophilic additions in this case as summarized in the upper right blue frame. For comparison, conventional nanoreactor simulations at *T* = 2000 K starting from the same initial geometries were conducted. However, these exhibit severe fragmentation of the initial reactants as shown in the lower left corner in black.

The way DNA formed on early Earth is still debated, as there is no conclusive evidence how this complex molecule, which plays an essential role for stable genetic information storage, could have formed from RNA in the absence of ribonucleotide reductases. Therefore, great effort has been put into finding synthesis pathways for deoxyribonucleotides under prebiotic conditions. In 2019, Trapp and co-workers^70^ presented a novel continuous path to deoxyribonucleosides starting from prebiotically available acetaldehyde (**42**) and d-glyceraldehyde (**90**) in an aqueous environment at 50 °C. The postulated reaction mechanism is summarized in the upper left of Figure 7 and involves an initial nucleophilic addition of acetaldehyde to N1/N9 of the pyrimidines and purines, respectively, followed by water elimination. The obtained enamine then initiates a nucleophilic attack at the carbonyl group and the compound finally cyclizes to yield the deoxyribonucleoside.

We have conducted investigations at *T*_target_ = 323.15 K starting from a 1:1:1:3 mixture of the nucleobase (A, G, T, C), acetaldehyde, d-glyceraldehyde, and water using the GaHRD and SaHRD approach with and without applying the ALPB implicit solvent model for water and compared the results to conventional computational nanoreactor simulations at 2000 K. Triplicates were performed for all simulations starting from three distinct initial configurations for each system. Full details on the employed parameters are given in the Supporting Information.

The obtained results clearly showcase the efficiency and advantages of HRD, as the nucleophilic addition of acetaldehyde at N9 leading to **A1** was successfully reproduced for adenine, when using water as an implicit solvent. The use of implicit solvation and performing the simulations at *T*_target_ = 323.15 K supported nucleophilic additions for all nucleobases at various positions. Therefore, additions of acetaldehyde and glyceraldehyde were mainly seen at N3, N7, and N9 for adenine and guanine, and at N3 for cytosine. For thymine, only additions at the carbonyl oxygen were observed and the expected enamine could not be retrieved. The simulations performed in vacuum lacked the expected enamines completely and in this case, nucleophilic additions at N3 and N7, as well as directly at the carbonyl oxygen predominated and yielded precursors for further cyclizations as shown in the lower right corner of Figure 7. Given the obtained glyceraldehyde adducts, e.g., **A4**, **A5**, or **G4**, we believe that a sequential exploration phase, where d-glyceraldehyde is added after the enamine has already been formed to first increase the chance of collision of the nucleobase and acetaldehyde, is recommended, as the number of atoms in *ab initio* MD simulations is still limited by the efficiency of the employed electronic structure method, therefore also statistically influencing the outcome.

In contrast, the conventional computational nanoreactor approach^6,8^ at 2000 K resulted in severe fragmentation of the reactants to hydrogen peroxide (**62**), formic acid (**94**), formaldehyde (**93**), acetonitrile (**20**), glycolaldehyde (**76**), and substituted oxiranes (**97**) among many others. Furthermore, highly functional aliphatic chains (**101**, **102**) were encountered. Only in one case, a nucleophilic addition was observed, at C8 of guanine, leading to **G5**.

Given the obtained results and the positive effect of implicit solvation and low thermostat temperatures on the studied system, the *ab initio* HRD approach delivers satisfactory outcomes at increased exploration rate and allows the use of experimentally defined reaction temperatures, as well as solvation environments for a better description of the molecular systems compared to the conventional *ab initio* nanoreactor method.

## Conclusion

We have investigated alternative reactivity enhancement techniques in the context of the computational nanoreactor approach first introduced by Wang et al.^6^ in combination with the smooth-step regulated external potential developed in our group^8^ to enable reaction space exploration at lower thermostat temperatures. Therefore, we have combined hyperdynamics approaches, namely aMD,^49^ GaMD,^50^ and SaMD,^51^ to elevate the PES with the smooth-step driven virtual piston to keep the molecules confined to the nanoreactor sphere within a new reaction space exploration approach termed *ab initio* hyperreactor dynamics (HRD).

Investigations on the optimal choice of the acceleration parameter (α or σ_0_) for a toy molecular system composed of 50 HCN molecules show positive correlation between the strength of the chosen boost potential Δ*V*(**x**) and the observed reactivity. Furthermore, the interplay between the strength of the periodic external potential given by *k*_conf_ and α/σ_0_ was also carefully examined to better understand the effect of the combined biases on the outcome. We recommend choosing *k*_conf_ between 0.75 and 1.00 kcal mol^–1^ Å^–2^ to achieve good confinement in the case of GaHRD/SaHRD at the recommended value for σ_0_^50^ of 10 *k*_B_*T* and *T* = 298.15 K. The choice of an ideal α in aHRD has proven to be difficult because it depends on the magnitude of the system’s initial potential energy.

As one of our main goals was to enable efficient and automated reaction space exploration at low equilibrium temperatures in order to avoid numerical problems and nonphysical structures, good temperature regulation was also ensured at the chosen friction constant γ = 7 ps^–1^ regardless of the chosen acceleration parameter and *k*_conf_. In addition, the influence of the thermostat temperature on the obtained reactivity for the three different acceleration approaches was investigated and revealed as expected a general increase in reactivity for higher equilibrium temperatures along with increased instability when surpassing *T* = 300 K. However, for all parameter investigations, the obtained results exhibit high variance depending on the initial configuration of the system, which makes averaging over multiple simulations employing the same parameters and different initial arrangements a necessity. Furthermore, out of the three tested hyperdynamics approaches, GaHRD provides increased stability independent of the chosen *k*_conf_, while a higher molecular variety is obtained with SaHRD.

To examine the performance of the HRD approach, we have chosen two experimentally investigated molecular systems stemming from origin of life hypotheses: the formation of glycinal and acetamide as precursors for polypeptides in an extraterrestrial synthesis^67^ at 10 K and a nonenzymatic synthesis of DNA nucleosides^70^ at 323.15 K. In the former case, we report the formation of the postulated precursors, glycinal, acetamide, and *N*-methylformamide, through various reaction pathways along with a multitude of prebiotically relevant molecules, which could aid the formation of more complex species under the experimentally described conditions. By including implicit water solvation within the ALPB model to the system composed of nucleobases, acetaldehyde, d-glyceraldehyde, and water necessary to model the nonenzymatic synthesis of DNA nucleosides, the postulated addition of acetaldehyde at N9 of adenine was achieved consistently. Additionally, the use of the HRD method supported nucleophilic additions at various positions of the nucleobases and hindered severe fragmentation of the reactants as observed in conventional computational nanoreactor simulations at 2000 K for the same molecular setups.

In conclusion, we report improved efficiency of the exploration phase within the computational nanoreactor framework by replacing the high thermostat temperatures by hyperdynamics derived bias potentials. The latter support the exploration of novel reaction pathways on an elevated PES with (partially) preserved topology under mild conditions. The presented approach represents a promising first-principles reaction space exploration method for modeling a wide variety of systems, regardless of the complexity of the reactants by employing a faster exploration protocol for the ideal acceleration strength than the conventional nanoreactor approach as demonstrated for the HCN system. Compared to the previously introduced metadynamics-based exploration protocol by Grimme,^52^ HRD enables an undirected exploration by using a CV-free enhanced sampling approach, therefore allowing for resampling of already visited regions on the PES. In the future, the method should benefit from the development of an adaptive version of the bias potential as already introduced and demonstrated for aMD,^54^ as well as from further automatization of the parameter choice.

## Supporting Information Available

Equations for determining *k* in GaMD and SaMD, details on the nanoreactor-processing package used for postprocessing of the simulations, all simulation parameters, additional figures on computational timings, obtained molecular species for all systems, and temperature and pressure regulation during the HCN test simulations, as well as initial geometries for the HCN system (PDF)
