# Supplementary material for: Exploring Chemical Space Using Ab Initio Hyperreactor Dynamics
Source: ACS Cent Sci. 2024 Jan 31;10(2):302–14. doi: 10.1021/acscentsci.3c01403 (PMC10906254; doi:10.1021/acscentsci.3c01403)
Supplement: Supplementary file 1 — oc3c01403_si_001.pdf [file oc3c01403_si_001.pdf]

# Supporting Information: Exploring Chemical Space Using Ab Initio Hyperreactor Dynamics

Alexandra Stan-Bernhardt,† Liubov Glinkina,† Andreas Hulm,† and Christian Ochsenfeld*,†,‡

†*Chair of Theoretical Chemistry, Department of Chemistry, University of Munich (LMU), Butenandtstr. 5, D-81377 München, Germany*

‡*Max Planck Institute for Solid State Research, Heisenbergstr. 1, D-70569 Stuttgart*

E-mail: christian.ochsenfeld@uni-muenchen.de

## Contents

**1 Relation Between $\sigma_0$ and $k$ for GaMD and SaMD** **S-2**

**2 Extension of the `nanoreactor-processing` Package** **S-5**

**3 Simulation Parameters** **S-5**

**4 Additional Results** **S-10**

**5 Overview of Obtained Molecular Species** **S-14**

**6 Initial Geometries for the HCN Test System** **S-17**

**References** **S-22**

# 1 Relation Between $\sigma_0$ and $k$ for GaMD and SaMD

In the following, we will describe the protocols in detail, that have been employed for the calculation of $k$ in GaMD and SaMD biasing potentials, as given by

$$\Delta V_{\mathrm{GaMD}} = \frac{1}{2} k \left(E - V(\mathbf{x})\right)^2 = \frac{1}{2} k_0 \frac{1}{V_{\mathrm{max}} - V_{\mathrm{min}}} \left(E - V(\mathbf{x})\right)^2, \tag{S1}$$

$$\Delta V_{\mathrm{SaMD}} = V_{\mathrm{max}} - V(\mathbf{x}) - \frac{1}{k} \ln \left[ \frac{C + \mathrm{e}^{k(V_{\mathrm{max}} - V_{\mathrm{min}})}}{C + \mathrm{e}^{k(V(\mathbf{x}) - V_{\mathrm{min}})}} \right]. \tag{S2}$$

To preserve the topology of the PES, the following condition must be fulfilled

$$V_{\mathrm{max}} \leq E \leq V_{\mathrm{min}} + \frac{1}{k}, \tag{S3}$$

which leads to

$$k \leq \frac{1}{V_{\mathrm{max}} - V_{\mathrm{min}}} = k_0 \frac{1}{V_{\mathrm{max}} - V_{\mathrm{min}}}. \tag{S4}$$

From Eq. S4 it becomes clear that $k_0$ defines the strength of the applied biasing potential $\Delta V$, where $0 < k_0 \leq 1$. Furthermore, as GaMD was developed with the goal to improve the accuracy of the reweighting to the original PES, the following condition additionally applies to ensure a small standard deviation for $\Delta V$

$$\sigma_{\Delta V} = k \left(E - V_{\mathrm{avg}}\right) \sigma_V \leq \sigma_0, \tag{S5}$$

which finally yields for $E = V_{\mathrm{max}}$ (the lower bound according to Eq. S3)

$$k_0 \leq \frac{\sigma_0}{\sigma_V} \frac{V_{\mathrm{max}} - V_{\mathrm{min}}}{V_{\mathrm{max}} - V_{\mathrm{avg}}}. \tag{S6}$$

Finally, we obtain the protocol for determining the maximal $k_0$ in GaMD starting from a

given $\sigma_0$ for the case where the boost energy $E$ is set to its lower bound as described above

$$k_0 = \min\left(1.0, k_\mathrm{o}\right) = \min\left(1.0, \frac{\sigma_0}{\sigma_V}\frac{V_\mathrm{max} - V_\mathrm{min}}{V_\mathrm{max} - V_\mathrm{avg}}\right). \tag{S7}$$

The equivalent formulations for the upper bound $E = V_\mathrm{min} + \frac{1}{k}$ are given in the original publication[S1] and will not be further detailed here, as we have only employed the lower bound for the boost energy in our simulation protocol for GaHRD.

In the case of SaMD, the same considerations apply for the upper and lower bound of $E$, as well as for $k_0$. However, the developers of this method[S2] base their derivation of the formulas for $\Delta V$ and the corresponding gradient by assuming a sigmoid form for the first derivative of the boost potential with respect to the potential energy, as shown in Fig. 1 of the main text,

$$\frac{\mathrm{d}}{\mathrm{d}V}\Delta V = F_{\Delta V} = \frac{1}{\mathrm{e}^{-k(V(\mathbf{x})-E)} + 1} - 1 \tag{S8}$$

so that

$$\underset{V \to V_\mathrm{min}}{F_{\Delta V}\left(V\right)} \to -1 \tag{S9}$$

$$\underset{V \to V_\mathrm{max}}{F_{\Delta V}\left(V\right)} \to 0. \tag{S10}$$

Normalization of $F_{\Delta V}$ to the interval of $(0, 1]$ yields

$$E = V_\mathrm{min} + \frac{1}{k}\ln\left(\frac{1}{C_0} - 1\right) \tag{S11}$$

and the final form for $F_{\Delta V}$

$$F_{\Delta V}(V) = \frac{1}{\mathrm{e}^{-k(V - V_\mathrm{min}) + \ln\left(\frac{1}{C_0} - 1\right)} + 1} - 1. \tag{S12}$$

By subsequent integration Eq. S2 is obtained where $C = \frac{1}{C_0} - 1$ and $C_0$ is a user-defined constant (set to $10^{-4}$ throughout this study) obtained as the limit for $F_{\Delta V}\left(V_{\min}\right) + 1$. The choice of $C_0$ in this work has been motivated by test calculations performed for the HCN system which showed a moderate slope of the sigmoidal curve corresponding to the biasing forces for the chosen value. Furthermore, we have decided to keep $C_0$ constant throughout the study to enable better comparison between GaHRD and SaHRD simulations by only varying $\sigma_0$. However, for the determination of $k$, further conditions stemming from Eq. S5 need to be taken into consideration to again ensure accurate reweighting as in GaMD. By applying $F_{\Delta V}\left(V_{\text{avg}}\right) \geq -\frac{\sigma_0}{\sigma_V}$ to Eq. S12, we obtain the final protocol for determining $k$ in SaHRD simulations

$$k = \begin{cases} k_0 & \text{if } \frac{\sigma_V}{\sigma_0} \leq 1, \\ \max\left(k_0, k_1\right) & \text{if } \frac{\sigma_V}{\sigma_0} > 1, \end{cases} \tag{S13}$$

where $k_1$ is defined as

$$k_1 = \max\left(0, \frac{\ln C + \ln\left(\frac{\sigma_0}{\sigma_V} - 1\right)}{V_{\text{avg}} - V_{\min}}\right) \tag{S14}$$

and $k_0$ is given in Eq. S7.

# 2 Extension of the `nanoreactor-processing` Package

To speed up the evaluation and post-processing of computational nanoreactor and HRD simulations, we have extended our `nanoreactor-processing` package[S3] by an interactive feature, which enables automatic drawing of reaction schemes containing molecular species of interest. For this purpose, the automatically generated reaction library is screened for a given molecular species specified by the user, which can be either a reactant or a product in the identified reaction schemes. The latter are then drawn using SMILES to reaction scheme conversion by RDKit[S4] and individually stored to enable quicker search for reactive events. In addition, we have introduced handling of sulfur and radical species into our parsing functions present in the `NanoSim` class to enable the evaluation of a wider range of molecular systems.

# 3 Simulation Parameters

Table S1: Piston-accelerated nanoreactor simulations for the homogeneous HCN toy model system.

| **Nanoreactor simulations of the HCN system** | | |
|---|---|---|
| Simulation | HCN-cNR-[1-6] | |
| Molecules | 50 HCN | |
| # Atoms | 150 | |
| Method | GFN2-xTB | |
| Basis Set | STO-$m$G | |
| $\Delta t$/fs | 0.5 | |
| Implicit Solvent | – | |
| $V_{\mathrm{Sphere}}$ | smooth-step spherical confinement | |
| $k_{\mathrm{conf}}$/kcal/(mol Å$^2$) | 1.00 | |
| $r_{\mathrm{min}}$/Å | 7 | |
| $r_{\mathrm{max}}$/Å | 15 | |
| $T_{\mathrm{target}}$/K | 298.15 | 2000.00 |
| $\gamma$/fs$^{-1}$ | $7 \times 10^{-3}$ | |
| $t_{\mathrm{total}}$/ps | 2.0 | |

Table S2: Homogeneous HCN toy model aHRD simulations performed with GFN2-xTB to investigate the interplay between $\alpha$ and $k_{\mathrm{conf}}$.

| **HCN aHRD simulations - GFN2-xTB** | | | | | | | | | |
|---|---|---|---|---|---|---|---|---|---|
| Simulation | HCN-aHRD-P[1-108] | | | | | | | | |
| Molecules | 50 HCN | | | | | | | | |
| # Atoms | 150 | | | | | | | | |
| Method | GFN2-xTB | | | | | | | | |
| Basis Set | STO-$m$G | | | | | | | | |
| $\Delta t$/fs | 0.5 | | | | | | | | |
| $\Delta V(\mathbf{x})$ | $\Delta V_{\mathrm{aMD}}$ | | | | | | | | |
| $t_{\mathrm{init}}$/ps | 0.5 | | | | | | | | |
| $t_{\mathrm{equil}}$/ps | 4.5 | | | | | | | | |
| $\alpha/E_{\mathrm{h}}$ | 0.0001 | 0.0005 | 0.010 | 0.025 | 0.050 | 0.10 | 0.50 | 1.00 | 2.00 |
| $V_{\mathrm{Sphere}}$ | smooth-step spherical confinement | | | | | | | | |
| $k_{\mathrm{conf}}$/kcal/(mol Å$^2$) | 0.25 | | 0.50 | | 0.75 | | 1.00 | | |
| $r_{\mathrm{min}}$/Å | 7 | | | | | | | | |
| $r_{\mathrm{max}}$/Å | 15 | | | | | | | | |
| $T_{\mathrm{target}}$/K | 298.15 | | | | | | | | |
| $\gamma$/fs$^{-1}$ | $7 \times 10^{-3}$ | | | | | | | | |
| $t_{\mathrm{total}}$/ps | 2.0 | | | | | | | | |

Table S3: GaHRD simulations performed for the homogeneous HCN toy model with GFN2-xTB to investigate the interplay between $\sigma_0$ and $k_{\mathrm{conf}}$.

| **HCN GaHRD simulations - GFN2-xTB** | | | | | | | | | |
|---|---|---|---|---|---|---|---|---|---|
| Simulation | HCN-GaHRD-P[1-108] | | | | | | | | |
| Molecules | 50 HCN | | | | | | | | |
| # Atoms | 150 | | | | | | | | |
| Method | GFN2-xTB | | | | | | | | |
| Basis Set | STO-$m$G | | | | | | | | |
| $\Delta t$/fs | 0.5 | | | | | | | | |
| $\Delta V(\mathbf{x})$ | $\Delta V_{\mathrm{GaMD}}$ | | | | | | | | |
| $t_{\mathrm{init}}$/ps | 0.5 | | | | | | | | |
| $t_{\mathrm{equil}}$/ps | 4.5 | | | | | | | | |
| $\sigma_0/E_{\mathrm{h}}$ | 0.0001 | 0.0005 | 0.002 | 0.005 | 0.008 | 0.010 | 0.012 | 0.015 | 0.016 |
| $V_{\mathrm{Sphere}}$ | smooth-step spherical confinement | | | | | | | | |
| $k_{\mathrm{conf}}$/kcal/(mol Å$^2$) | 0.25 | | 0.50 | | 0.75 | | 1.00 | | |
| $r_{\mathrm{min}}$/Å | 7 | | | | | | | | |
| $r_{\mathrm{max}}$/Å | 15 | | | | | | | | |
| $T_{\mathrm{target}}$/K | 298.15 | | | | | | | | |
| $\gamma$/fs$^{-1}$ | $7 \times 10^{-3}$ | | | | | | | | |
| $t_{\mathrm{total}}$/ps | 2.0 | | | | | | | | |

Table S4: Homogeneous HCN toy model SaHRD simulations performed with GFN2-xTB to investigate the interplay between $\sigma_0$ and $k_{\mathrm{conf}}$.

| **HCN SaHRD simulations - GFN2-xTB** | | | | | | | | | |
|---|---|---|---|---|---|---|---|---|---|
| Simulation | HCN-SaHRD-P[1-108] | | | | | | | | |
| Molecules | 50 HCN | | | | | | | | |
| # Atoms | 150 | | | | | | | | |
| Method | GFN2-xTB | | | | | | | | |
| Basis Set | STO-$m$G | | | | | | | | |
| $\Delta t/\mathrm{fs}$ | 0.5 | | | | | | | | |
| $\Delta V(\mathbf{x})$ | $\Delta V_{\mathrm{SaMD}}$ | | | | | | | | |
| $t_{\mathrm{init}}/\mathrm{ps}$ | 0.5 | | | | | | | | |
| $t_{\mathrm{equil}}/\mathrm{ps}$ | 4.5 | | | | | | | | |
| $\sigma_0/E_{\mathrm{h}}$ | 0.0001 | 0.0005 | 0.0008 | 0.001 | 0.005 | 0.008 | 0.010 | 0.012 | 0.015 |
| $V_{\mathrm{Sphere}}$ | smooth-step spherical confinement | | | | | | | | |
| $k_{\mathrm{conf}}/\mathrm{kcal/(mol\,Å^2)}$ | | 0.25 | | 0.50 | | 0.75 | | 1.00 | |
| $r_{\mathrm{min}}/\mathrm{Å}$ | 7 | | | | | | | | |
| $r_{\mathrm{max}}/\mathrm{Å}$ | 15 | | | | | | | | |
| $T_{\mathrm{target}}/\mathrm{K}$ | 298.15 | | | | | | | | |
| $\gamma/\mathrm{fs}^{-1}$ | $7 \times 10^{-3}$ | | | | | | | | |
| $t_{\mathrm{total}}/\mathrm{ps}$ | 2.0 | | | | | | | | |

Table S5: Homogeneous HCN model HRD simulations performed with GFN2-xTB to investigate the influence of the temperature on the outcome.

| **HRD HCN simulations at different temperatures** | | | |
|---|---|---|---|
| Simulation | HCN-aHRD-T[1-36] | HCN-GaHRD-T[1-36] | HCN-SaHRD-T[1-36] |
| Molecules | | 50 HCN | |
| # Atoms | | 150 | |
| Method | | GFN2-xTB | |
| Basis Set | | STO-$m$G | |
| $\Delta t/\mathrm{fs}$ | | 0.5 | |
| $\Delta V(\mathbf{x})$ | $\Delta V_{\mathrm{aMD}}$ | $\Delta V_{\mathrm{GaMD}}$ | $\Delta V_{\mathrm{SaMD}}$ |
| $t_{\mathrm{init}}/\mathrm{ps}$ | | 0.5 | |
| $t_{\mathrm{equil}}/\mathrm{ps}$ | | 4.5 | |
| $\alpha$, $\sigma_0/E_{\mathrm{h}}$ | [0.05, 0.10, 0.50] | [0.005, 0.008, 0.010] | [0.005, 0.010, 0.015] |
| $V_{\mathrm{Sphere}}$ | | smooth-step spherical confinement | |
| $k_{\mathrm{conf}}/\mathrm{kcal/(mol\,Å^2)}$ | | 1.00 | |
| $r_{\mathrm{min}}/\mathrm{Å}$ | | 7 | |
| $r_{\mathrm{max}}/\mathrm{Å}$ | | 15 | |
| $T_{\mathrm{target}}/\mathrm{K}$ | | [10.00, 100.00, 273.15, 323.15] | |
| $\gamma/\mathrm{fs}^{-1}$ | | $7 \times 10^{-3}$ | |
| $t_{\mathrm{total}}/\mathrm{ps}$ | | 2.0 | |

Table S6: aHRD parameters for the interstellar synthesis model of glycinal and acetamide.

| **HRD simulations of the prebiotic synthesis of glycinal and acetamide** | | | |
|---|---|---|---|
| Simulation | ExtSyn-[1-6] | ExtSyn-[7-12] | ExtSyn-[13-15] |
| Molecules | 13 $CH_3CHO$, 14 $NH_3$ | 12 $CH_3CHO$, 12 $NH_3$ <br> 2 $\cdot CH_2CHO$, 2 $\cdot NH_2$ | 7 $CH_3CHO$, 7 $NH_3$ <br> 7 $\cdot CH_2CHO$, 7 $\cdot NH_2$ |
| # Atoms | 147 | 150 | 140 |
| Method | | GFN2-xTB | |
| Basis Set | | STO-$m$G | |
| $\Delta t$/fs | | 0.5 | |
| $\Delta V(\mathbf{x})$ | | $\Delta V_{\mathrm{aMD}}$ | |
| $t_{\mathrm{init}}$/ps | | 0.5 | |
| $t_{\mathrm{equil}}$/ps | | 4.5 | |
| $\alpha/E_{\mathrm{h}}$ | [0.028, 0.08] | [0.025, 0.05] | 0.05 |
| $V_{\mathrm{Sphere}}$ | | smooth-step spherical confinement | |
| $k_{\mathrm{conf}}$/kcal/(mol Å$^2$) | | 1.00 | |
| $r_{\mathrm{min}}$/Å | 7 | 7 | 7 |
| $r_{\mathrm{max}}$/Å | 13 | 15 | 15 |
| $T_{\mathrm{target}}$/K | | 10.00 | |
| $\gamma$/fs$^{-1}$ | | $7 \times 10^{-3}$ | |
| $t_{\mathrm{total}}$/ps | | 2.0 | |

Table S7: GaHRD/SaHRD simulation parameters for the interstellar synthesis model of glycinal and acetamide.

| **HRD simulations of the prebiotic synthesis of glycinal and acetamide** | | | |
|---|---|---|---|
| Simulation | ExtSyn-[16-33] | ExtSyn-[34-51] | ExtSyn-[52-69] |
| Molecules | 13 $CH_3CHO$, 14 $NH_3$ | 12 $CH_3CHO$, 12 $NH_3$ <br> 2 $\cdot CH_2CHO$, 2 $\cdot NH_2$ | 7 $CH_3CHO$, 7 $NH_3$ <br> 7 $\cdot CH_2CHO$, 7 $\cdot NH_2$ |
| # Atoms | 147 | 150 | 140 |
| Method | | GFN2-xTB | |
| Basis Set | | STO-$m$G | |
| $\Delta t$/fs | | 0.5 | |
| $\Delta V(\mathbf{x})$ | | $\Delta V_{\mathrm{GaMD}}$, $\Delta V_{\mathrm{SaMD}}$ | |
| $t_{\mathrm{init}}$/ps | | 0.5 | |
| $t_{\mathrm{equil}}$/ps | | 4.5 | |
| $\sigma_0/E_{\mathrm{h}}$ | | [$3.167 \times 10^{-5}$, $3.167 \times 10^{-4}$, $3.167 \times 10^{-3}$] | |
| $V_{\mathrm{Sphere}}$ | | smooth-step spherical confinement | |
| $k_{\mathrm{conf}}$/kcal/(mol Å$^2$) | | 1.00 | |
| $r_{\mathrm{min}}$/Å | 7 | 7 | 7 |
| $r_{\mathrm{max}}$/Å | 13 | 15 | 15 |
| $T_{\mathrm{target}}$/K | | 10.00 | |
| $\gamma$/fs$^{-1}$ | | $7 \times 10^{-3}$ | |
| $t_{\mathrm{total}}$/ps | | 2.0 | |

Table S8: Piston-accelerated nanoreactor simulations for a non-enzymatic DNA nucleoside synthesis at 2000 K.

| **Nanoreactor simulations of a non-enzymatic DNA nucleoside synthesis** | |
|---|---|
| Simulation | NucSyn-[1-12] |
| Molecules | 5 A/T/G/C, 5 $CH_3CHO$, 5 D-glyceraldehyde, 15 $H_2O$ |
| # Atoms | 215/215/220/205 |
| Method | GFN2-xTB |
| Basis Set | STO-$m$G |
| $\Delta t$/fs | 0.5 |
| Implicit Solvent | – |
| $V_{\mathrm{Sphere}}$ | smooth-step spherical confinement |
| $t_{\mathrm{heating}}$/ps | 5.0 |
| $k_{\mathrm{conf}}$/kcal/(mol Å$^2$) | 1.00 |
| $r_{\mathrm{min}}$/Å | 10 |
| $r_{\mathrm{max}}$/Å | 20 |
| $T_{\mathrm{target}}$/K | 2000.00 |
| $\gamma$/fs$^{-1}$ | $7 \times 10^{-3}$ |
| $t_{\mathrm{total}}$/ps | 2.0 |

Table S9: GaHRD/SaHRD simulations for a non-enzymatic DNA nucleoside synthesis at 323.15 K with and without implicit water solvation.

| **HRD simulations of a non-enzymatic DNA nucleoside synthesis** | | |
|---|---|---|
| Simulation | NucSyn-[13-36] | NucSyn-[37-84] |
| Molecules | 5 A/T/G/C, 5 $CH_3CHO$, 5 D-glyceraldehyde, 15 $H_2O$ | |
| # Atoms | 215/215/220/205 | |
| Method | GFN2-xTB | |
| Basis Set | STO-$m$G | |
| $\Delta t$/fs | 0.5 | |
| Implicit Solvent | – | water (ALPB) |
| $\Delta V(\mathbf{r})$ | $\Delta V_{\mathrm{GaMD}}$ | $\Delta V_{\mathrm{SaMD}}$ |
| $t_{\mathrm{init}}$/ps | 0.5 | |
| $t_{\mathrm{equil}}$/ps | 4.5 | |
| $\sigma_0/E_{\mathrm{h}}$ | $1.000 \times 10^{-4}$ | $[1.000 \times 10^{-4}, 1.023 \times 10^{-2}]$ |
| $V_{\mathrm{Sphere}}$ | smooth-step spherical confinement | |
| $k_{\mathrm{conf}}$/kcal/(mol Å$^2$) | 1.00 | |
| $r_{\mathrm{min}}$/Å | 10 | |
| $r_{\mathrm{max}}$/Å | 20 | |
| $T_{\mathrm{target}}$/K | 323.15 | |
| $\gamma$/fs$^{-1}$ | $7 \times 10^{-3}$ | |
| $t_{\mathrm{total}}$/ps | 2.0 | |

# 4 Additional Results

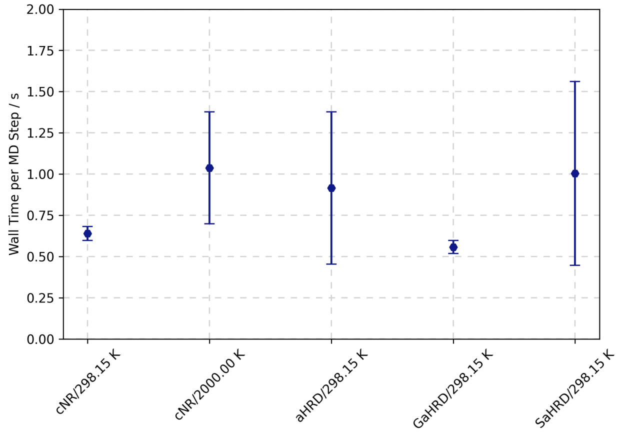


Figure S1: Obtained wall time per MD step for conventional nanoreactor simulations (cNR) at 298.15 and 2000 K and HRD simulations at 298.15 K of the HCN system on GFN2-xTB level of theory. The employed target temperature plays a significant role in destabilizing the simulations, besides inducing reactivity. Even though the chosen type of bias potential does not directly influence the time of computation, the higher mean timings and standard deviation obtained for aHRD and SaHRD originate from the difficulty of identifying optimal acceleration parameters for these methods. However, the induced reactivity for all three HRD variants at 298.15 K is comparable with the cNR/2000.00 K simulations, while no reactivity was observed for the cNR/298.15 K simulations. For the piston-accelerated nanoreactor simulations the results were obtained as a mean over triplicates, while the mean for the HRD simulations was done over triplicates for each of the acceleration parameters presented in Table S5. The IQR method[S5] was used to remove outliers prior to analysis.

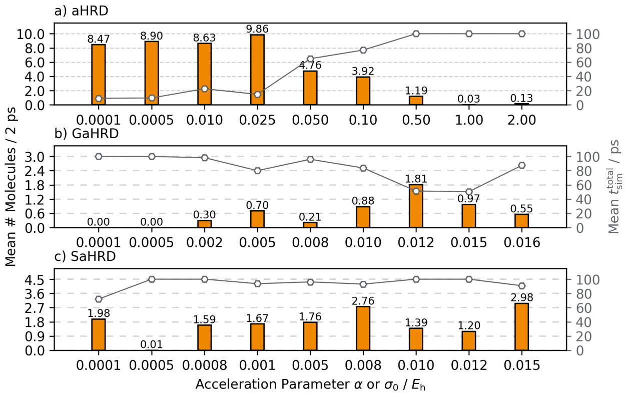


Figure S2: Obtained number of new molecular species every 2 ps during different types of HRD simulations at $k_{\text{conf}} = 1.00\,\text{kcal}\,\text{mol}^{-1}\,\text{Å}^{-2}$ shown in orange. All numbers are reported as the average over triplicates. The total simulation length is depicted in dark grey to enable interpretation of the obtained quantitative results and to highlight differences in the simulation's stability behavior.

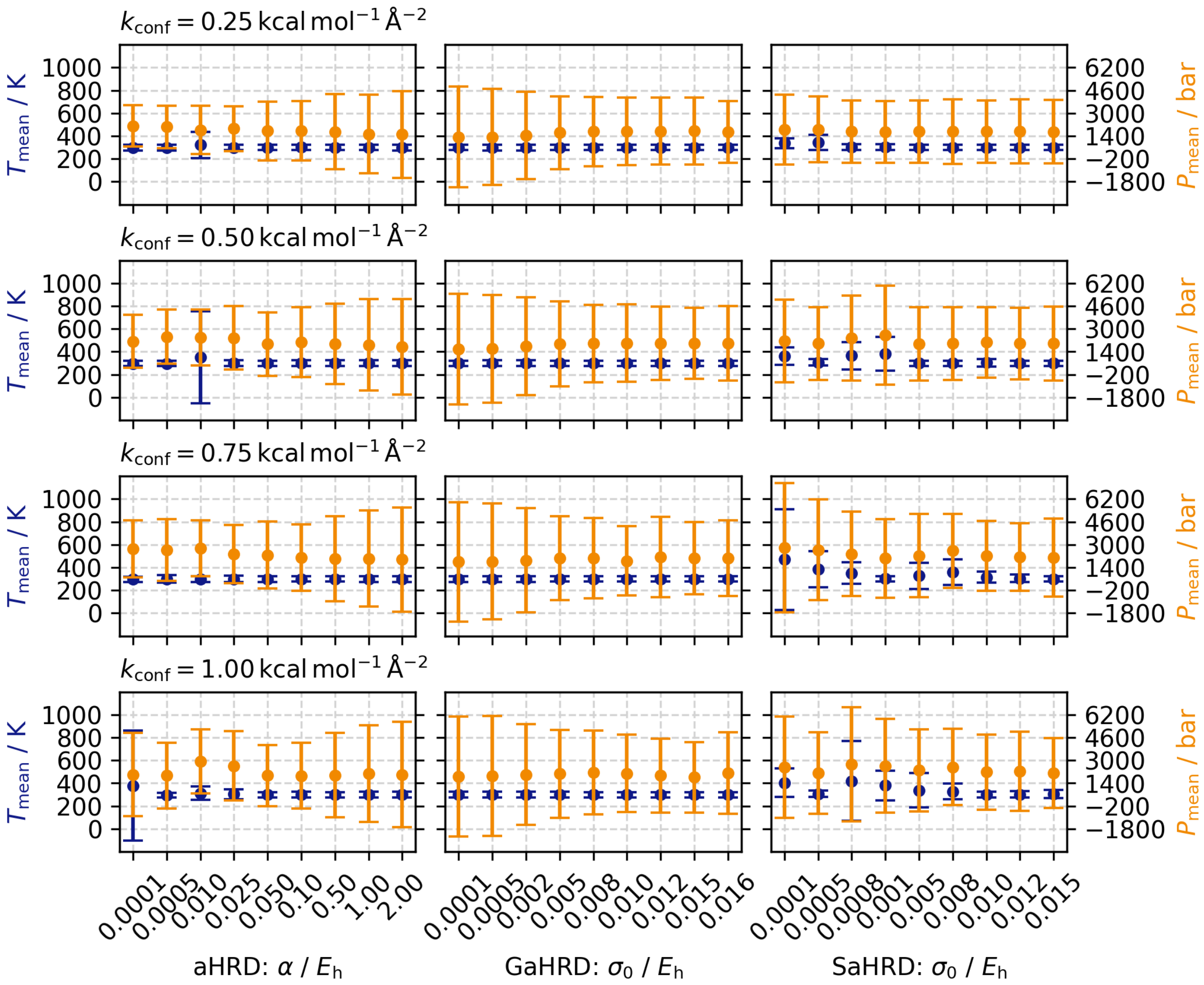


Figure S3: Temperature and pressure regulation in HRD simulations employing different bias potentials for reaction enhancement. While the Langevin thermostat provides excellent temperature control, pressure fluctuations are present and correlate with increasing $k_{conf}$ and decreasing $\alpha$; $\sigma_0$. This is attributed to larger dissipation of the molecules and resulting harsher spherical confinement based on the increasing radial coordinate. All results are reported as a mean over triplicates for every $k_{conf}/\alpha$; $\sigma_0$ setup over a total simulation time of 100 ps after data curation by the IQR method[S5] to remove eventual outliers.

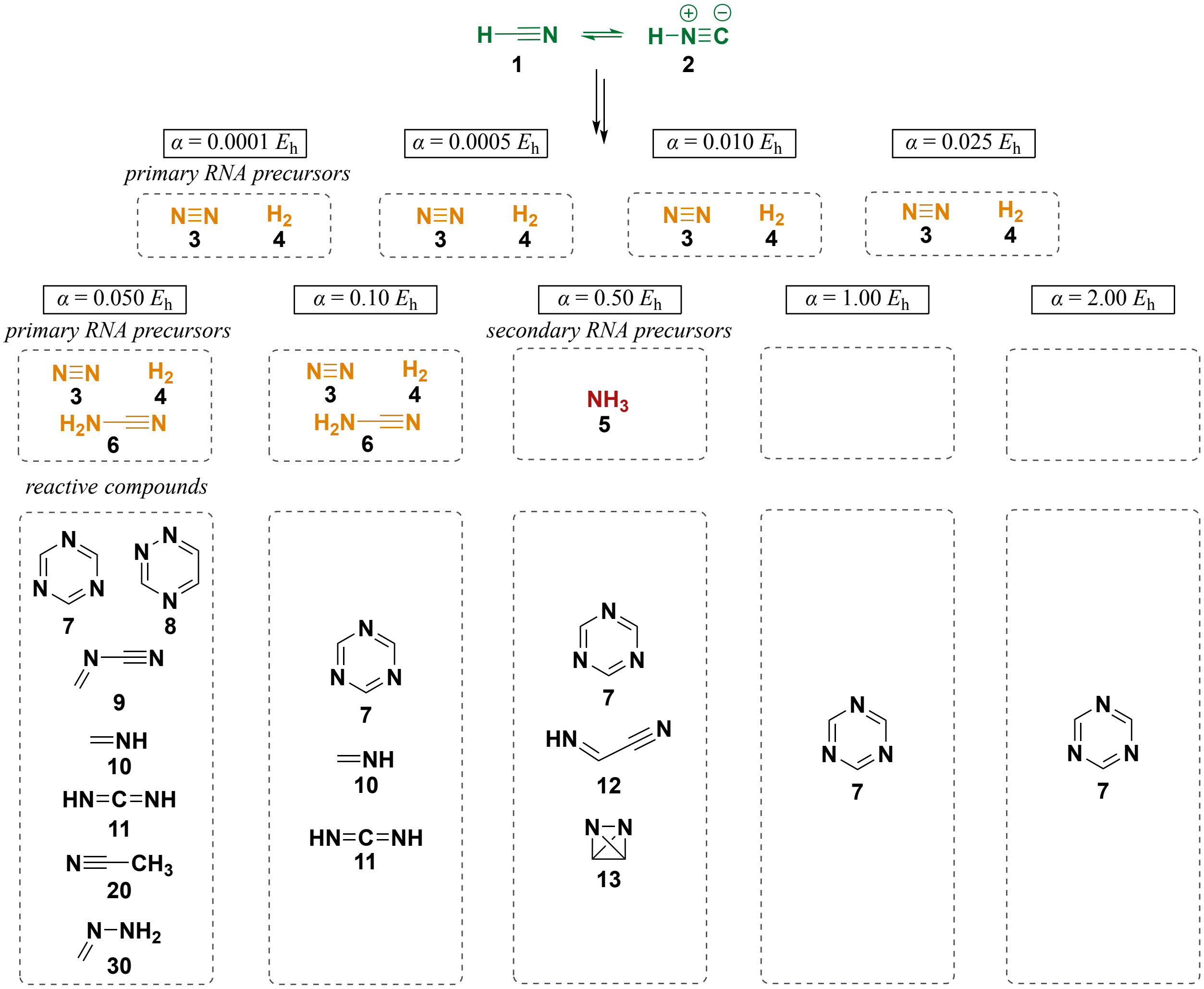


Figure S4: Relevant prebiotic and reactive compounds obtained in HCN aHRD simulations at different acceleration strength $\alpha$ and $k_{\mathrm{conf}} = 1.00\,\mathrm{kcal\,mol^{-1}\,\AA^{-2}}$. Initial compounds are given in green. Primary and secondary RNA precursors are shown in orange and red, respectively. The classification was done according to Benner *et al.*[S6]

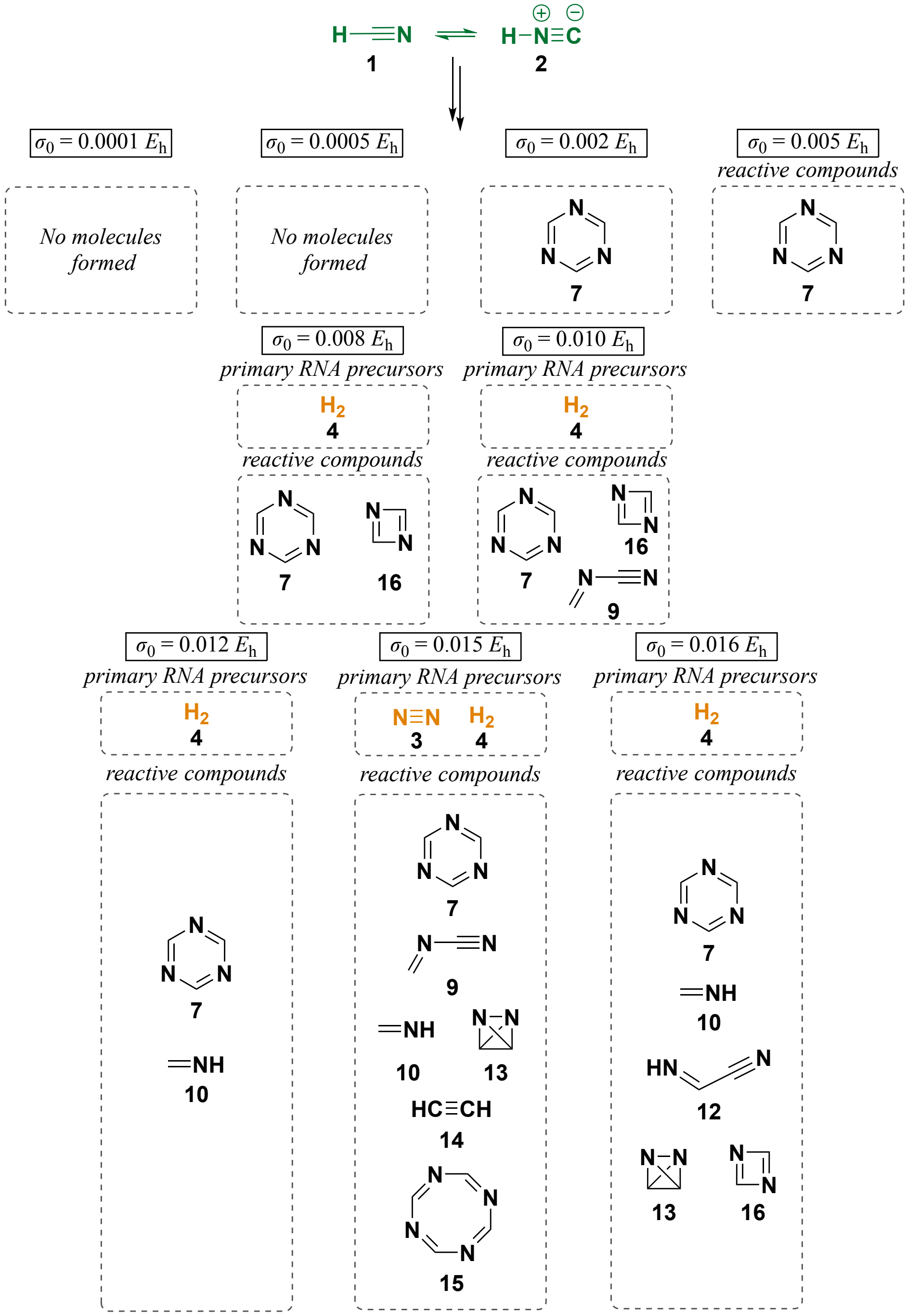


Figure S5: Relevant prebiotic and reactive compounds obtained in HCN GaHRD simulations at different acceleration strength $\sigma_0$ and $k_{\mathrm{conf}} = 1.00\,\mathrm{kcal\,mol^{-1}\,Å^{-2}}$. Initial compounds are given in green. Primary and secondary RNA precursors are shown in orange and red, respectively. The classification was done according to Benner *et al.*[S6]

# 5 Overview of Obtained Molecular Species

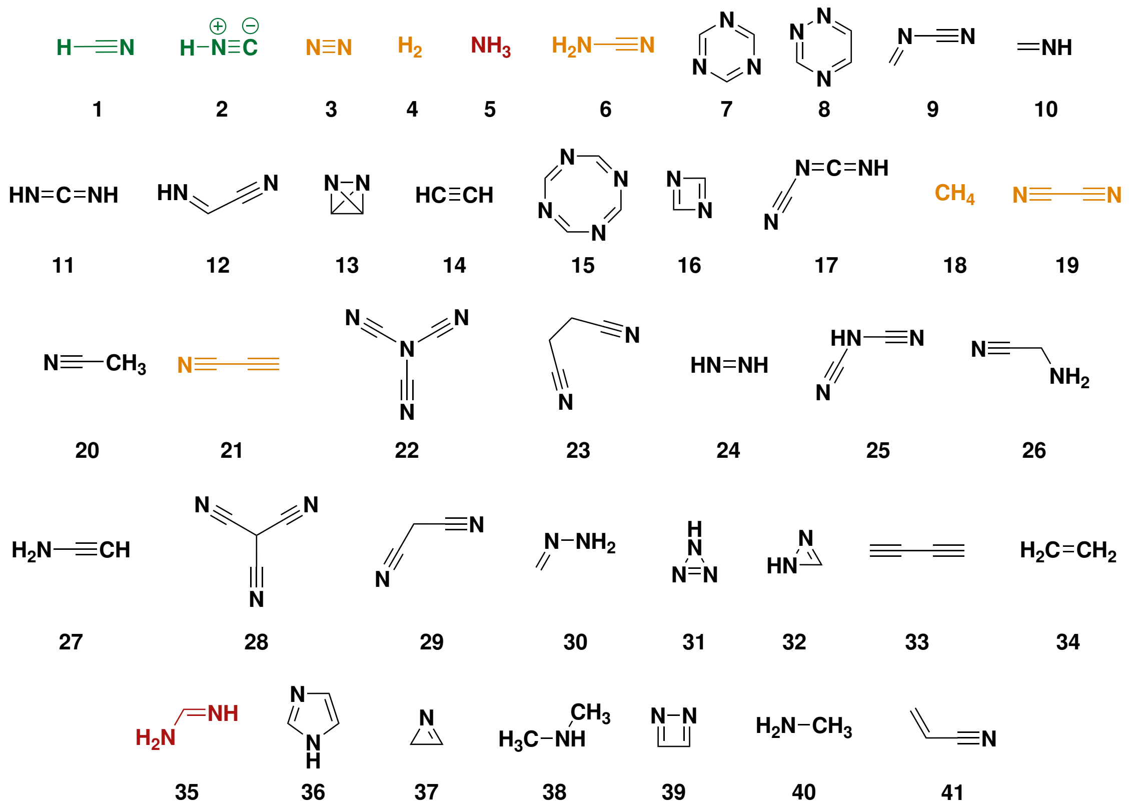

Figure S6: Selection of relevant persistent molecular species found overall for the HCN toy model system. Initial compounds are given in green. Primary and secondary RNA precursors are shown in orange and red, respectively. The classification was done according to Benner *et al.*[S6]

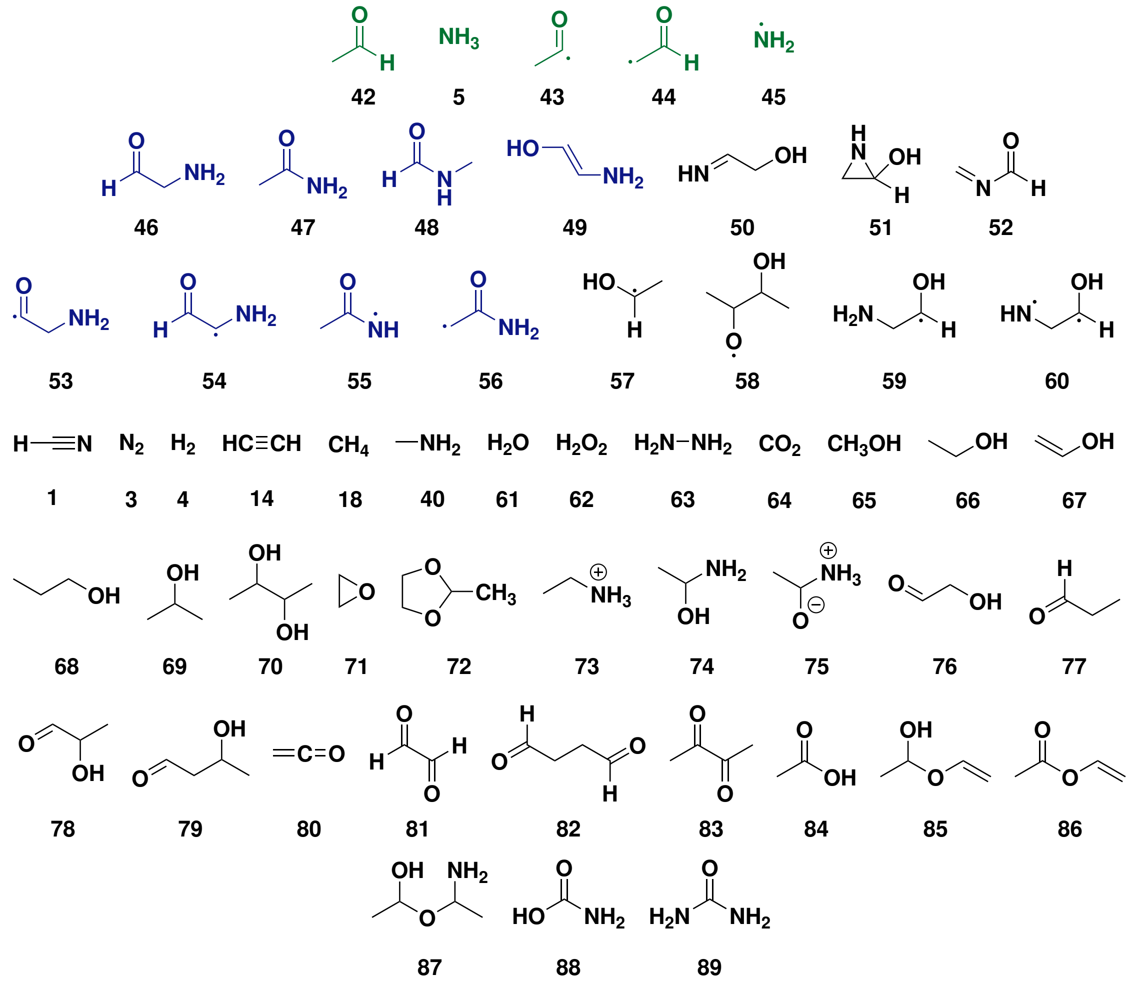


Figure S7: Selection of relevant persistent molecular species obtained in aHRD, GaHRD, and SaHRD simulations of the prebiotic synthesis of glycinal and acetamide in interstellar ices. Initial compounds are given in green, while precursors matching the experimental data[S7] are depicted in dark blue.

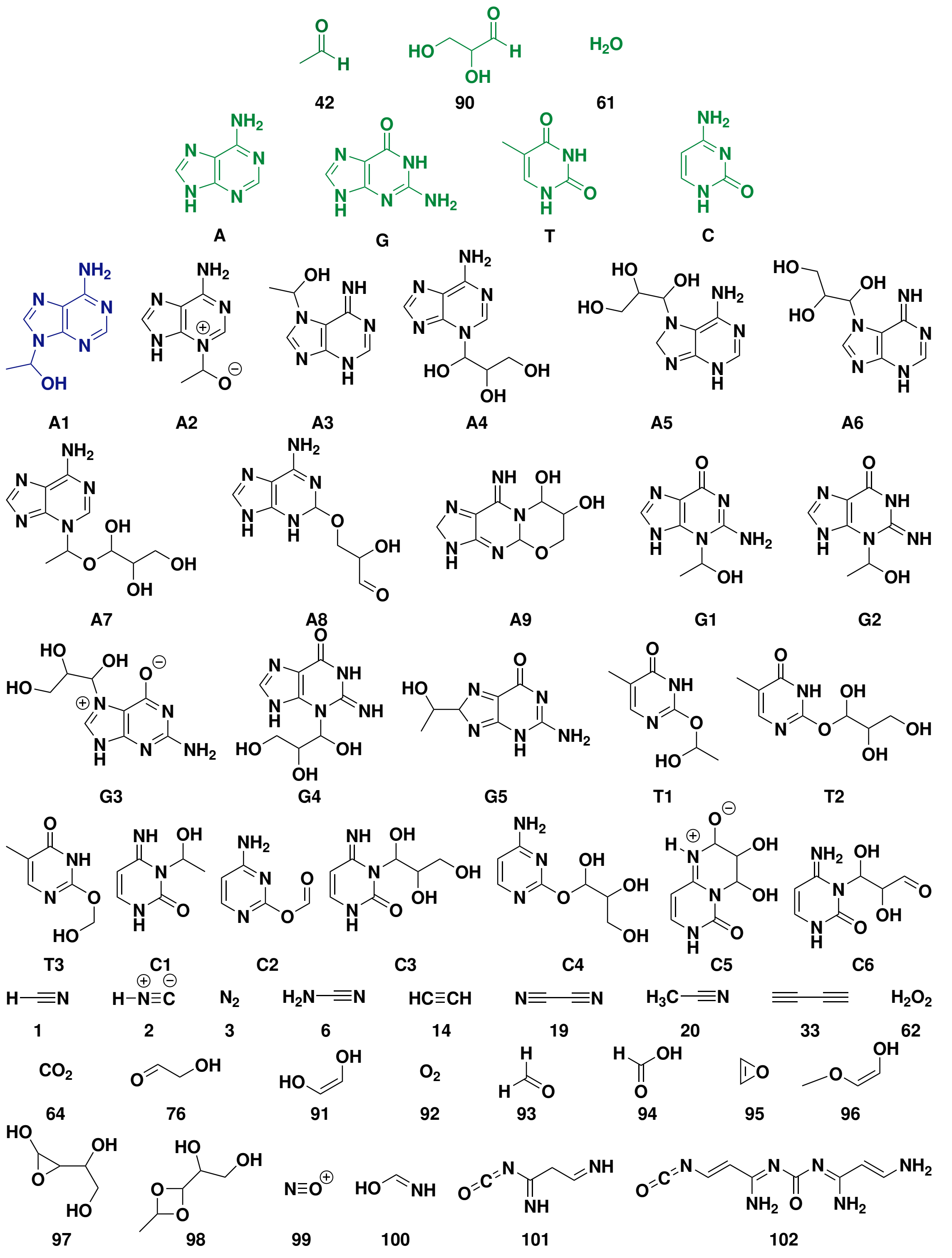


Figure S8: Selection of relevant persistent molecular species obtained in GaHRD and SaHRD simulations of the prebiotic non-enzymatic synthesis of DNA nucleosides.[S8] Initial compounds are given in green, while precursors matching the experimental data are depicted in dark blue.

# 6 Initial Geometries for the HCN Test System

| | | HCN_CONFIG_01 | | | | HCN_CONFIG_02 | |
|---|---|---|---|---|---|---|---|
| C | -4.8833818819e-01 | 2.4724040100e-01 | 2.9119679295e+00 | C | -3.8509872521e-01 | -3.9872415527e-01 | 3.0083466879e+00 |
| N | 5.2149089512e-01 | -2.6402589409e-01 | 3.0940091456e+00 | N | 4.1124255709e-01 | 4.2579366473e-01 | 2.9910859541e+00 |
| H | -1.4276568664e+00 | 7.2281858499e-01 | 2.7426281310e+00 | H | -1.1258346298e+00 | -1.1656807216e+00 | 3.0244122004e+00 |
| C | -2.0021721978e+00 | 1.5437075549e+00 | 1.7383540790e-01 | C | -1.9555474903e+00 | 1.7866776647e+00 | -4.2898515779e-01 |
| N | -2.4362941841e+00 | 2.5420084324e+00 | -1.8563707414e-01 | N | -2.4860841012e+00 | 2.2825437616e+00 | 4.5810825600e-01 |
| H | -1.5983482948e+00 | 6.1511170980e-01 | 5.0820970035e-01 | H | -1.4620419728e+00 | 1.3254302204e+00 | -1.2541356017e+00 |
| C | -4.4343638604e-01 | 3.2294320973e-01 | -3.0801467540e+00 | C | 3.2555086087e-01 | 1.3699319757e-01 | -3.4273185558e+00 |
| N | 4.7354056807e-01 | -3.4486832764e-01 | -2.9144119583e+00 | N | -3.4765185878e-01 | -1.4629316142e-01 | -2.5436704678e+00 |
| H | -1.2963836142e+00 | 9.4413920203e-01 | -3.2343113443e+00 | H | 9.5174470156e-01 | 4.0049319623e-01 | -4.2492791269e+00 |
| C | 1.6097784468e-01 | -4.8720758790e-01 | -5.7900796647e+00 | C | 3.4470890468e-01 | 2.1191601659e-01 | -6.3789710019e+00 |
| N | -1.7190595763e-01 | 5.2028427342e-01 | -6.2241712793e+00 | N | -3.6811058838e-01 | -2.2630361382e-01 | -5.5953008340e+00 |
| H | 4.7061031706e-01 | -1.4243634439e+00 | -5.3863015210e+00 | H | 1.0077528803e+00 | 6.1955007722e-01 | -7.1079273207e+00 |
| C | 4.2322246614e+00 | -3.6669437136e+00 | -3.3494415132e+00 | C | 4.0921350901e+00 | -3.9926385082e+00 | -3.0801464306e+00 |
| N | 3.4035307370e+00 | -3.3422998038e+00 | -2.6268353149e+00 | N | 3.5531310375e+00 | -2.9944933989e+00 | -2.9144117680e+00 |
| H | 5.0030668638e+00 | -3.9689108564e+00 | -4.0215935045e+00 | H | 4.5935109885e+00 | -4.9210902244e+00 | -3.2343209260e+00 |
| C | -9.7241508602e-01 | 5.9438701468e+00 | 3.2507396099e-01 | C | -3.3138612668e-01 | 5.5208203043e+00 | 2.4882488820e-01 |
| N | -4.6288893731e-02 | 6.0124339570e+00 | -3.4714347871e-01 | N | -7.3083614406e-01 | 6.4642042666e+00 | -2.6571703381e-01 |
| H | -1.8338758486e+00 | 5.8800826135e+00 | 9.5036322269e-01 | H | 4.0165999986e-02 | 4.6432951503e+00 | 7.2743618871e-01 |
| C | -3.6021108538e+00 | -3.8553761015e+00 | 2.7968546353e+00 | C | -2.7699030059e+00 | -3.7878866153e+00 | 2.7969717113e+00 |
| N | -2.6910606808e+00 | -4.4101786821e+00 | 3.2169363953e+00 | N | -3.5797669008e+00 | -4.4822511751e+00 | 3.2168113667e+00 |
| H | -4.4495604308e+00 | -3.3393178379e+00 | 2.4061076087e+00 | H | -2.0165886058e+00 | -3.1419916328e+00 | 2.4064499552e+00 |
| C | 1.9793776237e-01 | 2.4985959051e-02 | 6.5172504146e+00 | C | 4.1659751229e-02 | -4.1252674866e-02 | 5.4487157455e+00 |
| N | -2.1137488001e-01 | -2.6682339602e-02 | 5.4476334018e+00 | N | -4.4488592491e-02 | 4.4052687511e-02 | 6.5887106860e+00 |
| H | 5.7866105846e-01 | 7.3049574330e-02 | 7.5121925318e+00 | H | 1.2180112491e-01 | -1.2059203043e-01 | 4.3883135658e+00 |
| C | 8.2483677837e-01 | 4.8752409058e-01 | 9.2265911443e+00 | C | 1.1742740524e+00 | -3.6142367149e-01 | 8.8085132484e+00 |
| N | 7.4122083353e-01 | -5.2062209061e-01 | 8.6872057001e+00 | N | 3.6806133407e-01 | 3.8596049859e-01 | 9.1336669988e+00 |
| H | 9.0262521519e-01 | 1.4252856116e+00 | 9.7283106322e+00 | H | 1.9241931953e+00 | -1.0566266080e+00 | 8.5060495119e+00 |
| C | -5.2377918435e+00 | 4.8566431887e+00 | 6.3142286269e+00 | C | -4.7449447428e+00 | 4.8131813475e+00 | 5.4287080484e+00 |
| N | -4.8286173875e+00 | 4.3610465923e+00 | 5.3648881797e+00 | N | -5.3549230327e+00 | 4.4074588844e+00 | 6.3105259293e+00 |
| H | -5.6183890067e+00 | 5.3176466304e+00 | 7.1972868253e+00 | H | -4.1775562402e+00 | 5.1905887181e+00 | 4.6084616711e+00 |
| C | 8.2041249087e-01 | -8.1867741389e+00 | 2.6306718126e+00 | C | 1.0276481187e+00 | -8.7154749830e+00 | 2.7780669143e+00 |
| N | 6.4915212876e-01 | -8.6369996490e+00 | 3.6709861328e+00 | N | 4.2784757233e-01 | -8.0724043016e+00 | 3.5135846975e+00 |
| H | 9.7970813321e-01 | -7.7679729409e+00 | 1.6629938648e+00 | H | 1.5855624555e+00 | -9.3136564096e+00 | 2.0939026580e+00 |
| C | 4.9258500257e+00 | 7.0802633042e+00 | 6.1192266988e-01 | C | 5.1560007501e+00 | 7.2175559550e+00 | 9.5497385624e-01 |
| N | 6.0455931373e+00 | 7.1855499893e+00 | 3.8971311983e-01 | N | 5.7998169743e+00 | 7.0389359590e+00 | 2.3371702551e-02 |
| H | 3.8842863242e+00 | 6.9823153605e+00 | 8.1861425614e-01 | H | 4.5571445146e+00 | 7.3837009512e+00 | 1.8215401867e+00 |
| C | -9.0834553352e+00 | -1.7892795757e+00 | -2.2317779819e+00 | C | -8.3539970511e+00 | -1.8585455074e+00 | -2.4063025517e+00 |
| N | -8.1412092152e+00 | -1.2451286833e+00 | -1.8707252684e+00 | N | -8.9201902082e+00 | -1.1711603478e+00 | -1.6843510592e+00 |
| H | -9.9599216698e+00 | -2.2954336505e+00 | -2.5676127218e+00 | H | -7.8273406588e+00 | -2.4979328493e+00 | -3.0778582286e+00 |
| C | 6.0256679727e+00 | -4.3026569882e+00 | -4.4381841132e+00 | C | 6.9095961372e+00 | -3.8697748945e+00 | -4.2505214282e+00 |
| N | 7.1126185856e+00 | -4.0229627392e+00 | -4.6718430495e+00 | N | 6.1686814019e+00 | -4.4852340777e+00 | -4.8722467335e+00 |
| H | 5.0146055985e+00 | -4.5628284101e+00 | -4.2208505850e+00 | H | 7.5987742552e+00 | -3.2972757570e+00 | -3.6722062763e+00 |
| C | -1.6123385650e+00 | 6.0474224056e+00 | -7.0579831598e+00 | C | -1.1659665287e+00 | 5.9067574153e+00 | -7.1892883722e+00 |
| N | -1.3458958436e+00 | 4.9536978213e+00 | -6.8410458043e+00 | N | -1.8225729785e+00 | 5.1039120604e+00 | -6.7008272043e+00 |
| H | -1.8601905568e+00 | 7.0647811255e+00 | -7.2597770671e+00 | H | -5.5519987821e-01 | 6.6535532641e+00 | -7.6436342916e+00 |
| C | 5.3910010634e-01 | 1.1940706500e+00 | -9.1326772135e+00 | C | 8.7841066582e-01 | 6.0789179418e-01 | -8.7857273842e+00 |
| N | 1.7191686105e-01 | 1.6435578160e-01 | -8.7874950000e+00 | N | -1.9042823755e-01 | 7.9032995672e-01 | -9.1579997601e+00 |
| H | 8.8065910012e-01 | 2.1518888541e+00 | -9.4537564833e+00 | H | 1.8726203040e+00 | 4.3819004322e-01 | -8.4394346368e+00 |
| C | -1.5488825184e+00 | -2.1666600267e-01 | -1.1868401498e+01 | C | -1.0590745060e+00 | 5.4790167342e-01 | -1.2037888851e+01 |
| N | -5.0870565010e-01 | 2.3137492101e-01 | -1.2046104677e+01 | N | -1.0317665028e+00 | -5.8509857316e-01 | -1.1865112347e+01 |
| H | -2.5164404256e+00 | -6.3341776938e-01 | -1.1703114424e+01 | H | -1.0844815673e+00 | 1.6017989420e+00 | -1.2198591011e+01 |
| C | 5.7346806839e+00 | -5.4958866248e+00 | -9.1338695629e+00 | C | 5.9886435092e+00 | -5.2625564089e+00 | -9.1862735241e+00 |

| | | | | | | | |
|---|---|---|---|---|---|---|---|
| N | 5.2808161705e+00 | -4.5787517792e+00 | -9.6507425500e+00 | N | 5.0096123069e+00 | -4.8279214841e+00 | -9.5947798197e+00 |
| H | 6.1568619248e+00 | -6.3489808021e+00 | -8.6530749288e+00 | H | 6.8993175232e+00 | -5.6668568088e+00 | -8.8062943371e+00 |
| C | -1.1324911495e+00 | 9.1645245799e+00 | -6.9517037474e+00 | C | -9.5162104958e-01 | 1.0112605171e+01 | -7.2248437579e+00 |
| N | -5.2752925235e-01 | 1.0004440974e+01 | -7.4445004202e+00 | N | -7.2068003562e-01 | 8.9919957412e+00 | -7.1528176486e+00 |
| H | -1.6952261784e+00 | 8.3832550566e+00 | -6.4933181293e+00 | H | -1.1664264033e+00 | 1.1154977831e+01 | -7.2918366917e+00 |
| C | -6.3647133150e+00 | -8.5965791181e+00 | -4.5790544981e+00 | C | -6.7718745042e+00 | -9.1633003058e+00 | -5.0775789124e+00 |
| N | -6.8868172734e+00 | -8.6676715110e+00 | -5.5972098569e+00 | N | -6.4520133639e+00 | -8.0624762572e+00 | -5.0648403434e+00 |
| H | -5.8790519656e+00 | -8.5304567049e+00 | -3.6319900674e+00 | H | -7.0694052823e+00 | -1.0187265584e+01 | -5.0894406850e+00 |
| C | 1.1945107048e+01 | 1.8616447546e+00 | -2.8326602522e+00 | C | 1.1393561253e+01 | 2.2656565312e+00 | -3.4952890436e+00 |
| N | 1.0904036640e+01 | 2.1971068412e+00 | -3.1760617665e+00 | N | 1.1493027146e+01 | 1.7656657180e+00 | -2.4684479025e+00 |
| H | 1.2913488139e+01 | 1.5495920347e+00 | -2.5132368376e+00 | H | 1.1301028615e+01 | 2.7307434358e+00 | -4.4504340787e+00 |
| C | -1.0228417120e+01 | 6.9180567539e+00 | -7.5898478908e-01 | C | -1.0324421891e+01 | 5.9493477601e+00 | -7.8219408224e-01 |
| N | -9.9474255626e+00 | 5.8882206462e+00 | -1.1770378267e+00 | N | -9.8449024068e+00 | 6.9226952661e+00 | -1.1522517852e+00 |
| H | -1.0489778928e+01 | 7.8759917653e+00 | -3.7011376289e-01 | H | -1.0770461472e+01 | 5.0439533391e+00 | -4.3798514908e-01 |
| C | 2.8027466769e+00 | -1.1921082677e+01 | 7.7176403410e-01 | C | 2.9756586882e+00 | -1.1516105181e+01 | 5.0713963786e-01 |
| N | 3.4244584043e+00 | -1.1142315216e+01 | 1.3385821500e+00 | N | 3.2398081132e+00 | -1.1574786274e+01 | 1.6211722302e+00 |
| H | 2.2244477148e+00 | -1.2645486962e+01 | 2.4452699312e-01 | H | 2.7299481238e+00 | -1.1461533330e+01 | -5.2911244892e-01 |
| C | 5.6406135609e+00 | 9.9778552699e+00 | 2.6819179155e+00 | C | 5.4528995327e+00 | 9.7773698090e+00 | 3.1579386314e+00 |
| N | 5.0446861389e+00 | 1.0655971244e+01 | 3.3885410399e+00 | N | 5.2451440947e+00 | 1.0870067323e+01 | 2.8802034549e+00 |
| H | 6.1949238159e+00 | 9.3470771182e+00 | 2.0246286183e+00 | H | 5.6461372829e+00 | 8.7609604277e+00 | 3.4162820019e+00 |
| C | -9.7755815297e+00 | -3.9484329999e+00 | 5.1515678211e+00 | C | -1.0140826892e+01 | -4.2585363469e+00 | 4.7938629409e+00 |
| N | -1.0775854895e+01 | -3.5312281286e+00 | 4.7778295476e+00 | N | -1.0385813774e+01 | -3.2000725844e+00 | 5.1598177166e+00 |
| H | -8.8451486560e+00 | -4.3365204721e+00 | 5.4992069115e+00 | H | -9.9129370333e+00 | -5.2431012962e+00 | 4.4534687159e+00 |
| C | 9.2419105210e+00 | -3.9064097815e+00 | 6.3741685753e+00 | C | 9.1633485933e+00 | -4.2640596902e+00 | 6.6885328129e+00 |
| N | 8.9198238887e+00 | -3.5842792272e+00 | 7.4262042750e+00 | N | 9.0037208261e+00 | -3.2023489628e+00 | 7.0904983756e+00 |
| H | 9.5415199158e+00 | -4.2060551190e+00 | 5.3955894839e+00 | H | 9.3118187835e+00 | -5.2516452632e+00 | 6.3146324326e+00 |
| C | -3.4808263315e+00 | 7.9511746365e+00 | 8.9607867104e+00 | C | -3.1821003911e+00 | 7.1199279316e+00 | 8.4080539347e+00 |
| N | -3.4812752057e+00 | 6.8916130407e+00 | 8.5230470279e+00 | N | -3.8002808531e+00 | 7.7792934468e+00 | 9.1133035643e+00 |
| H | -3.4803972722e+00 | 8.9367611385e+00 | 9.3679589327e+00 | H | -2.6070784097e+00 | 6.5065901899e+00 | 7.7520534079e+00 |
| C | -1.9752343844e+00 | -4.9976219134e+00 | 1.0421317802e+01 | C | -1.7774212202e+00 | -5.0234501409e+00 | 1.0858068509e+01 |
| N | -1.4408768288e+00 | -5.9817172886e+00 | 1.0666889179e+01 | N | -1.6521185258e+00 | -5.9541354382e+00 | 1.0200488766e+01 |
| H | -2.4722829457e+00 | -4.0822312457e+00 | 1.0192904122e+01 | H | -1.8939866651e+00 | -4.1577416249e+00 | 1.1469731340e+01 |
| C | -4.7671294508e-01 | -1.8268823007e-01 | 1.1869258870e+01 | C | -1.3586395404e+00 | -8.3435780260e-01 | 1.1880851221e+01 |
| N | -1.3621512944e+00 | -8.8932389917e-01 | 1.2045190581e+01 | N | -4.2035065755e-01 | -1.9341361400e-01 | 1.2032810182e+01 |
| H | 3.4690573503e-01 | 4.7460746214e-01 | 1.1705598250e+01 | H | -2.2314255547e+00 | -1.4305426565e+00 | 1.1739506434e+01 |
| C | 2.1237611805e+00 | 2.5440739298e-01 | 1.4878439276e+01 | C | 3.0060374801e+00 | 3.8248875730e-01 | 1.4772762136e+01 |
| N | 3.1183368036e+00 | -2.7167842709e-01 | 1.4658575576e+01 | N | 2.1761623505e+00 | -4.0845529485e-01 | 1.4771427185e+01 |
| H | 1.1986195888e+00 | 7.4375486882e-01 | 1.5082944139e+01 | H | 3.7779788398e+00 | 1.1182028119e+00 | 1.4773996147e+01 |
| C | -6.7680876444e+00 | 6.3151510791e+00 | 1.1883304465e+01 | C | -6.6563052683e+00 | 5.8013099566e+00 | 1.2667153432e+01 |
| N | -6.1651313499e+00 | 5.5249284533e+00 | 1.2454512759e+01 | N | -6.2845033083e+00 | 6.0736530763e+00 | 1.1617448934e+01 |
| H | -7.3289566364e+00 | 7.0501985965e+00 | 1.1351984150e+01 | H | -7.0021461638e+00 | 5.5479933817e+00 | 1.3643572883e+01 |
| C | 1.0889448000e+00 | -1.1249421964e+01 | 9.8408294210e+00 | C | 9.4112512278e-01 | -1.0461799582e+01 | 9.9223625184e+00 |
| N | 8.1620901545e-01 | -1.0537468868e+01 | 1.0696998205e+01 | N | 9.7406288229e-01 | -1.1378561913e+01 | 1.0609929926e+01 |
| H | 1.3426279408e+00 | -1.1911664015e+01 | 9.0444309745e+00 | H | 9.1049650728e-01 | -9.6090492827e+00 | 9.2827931554e+00 |
| C | 7.6780351317e+00 | 9.6504718613e+00 | 7.9739248614e+00 | C | 7.8268429048e+00 | 9.9959961815e+00 | 8.9124653347e+00 |
| N | 7.3678521709e+00 | 9.9989478628e+00 | 9.0211297189e+00 | N | 7.2089421073e+00 | 9.6299657951e+00 | 8.0188729405e+00 |
| H | 7.9665738135e+00 | 9.3263271032e+00 | 6.9998377192e+00 | H | 8.4016124145e+00 | 1.0336473484e+01 | 9.7436604819e+00 |
| C | -1.2674732293e+01 | -2.1061407627e+00 | 6.7040287429e+00 | C | -1.3716935801e+01 | -2.2500469447e+00 | 6.6887852268e+00 |
| N | -1.3711267260e+01 | -2.5703937005e+00 | 6.8600783445e+00 | N | -1.2598309161e+01 | -2.4167166882e+00 | 6.8763555965e+00 |
| H | -1.1710570949e+01 | -1.6742957073e+00 | 6.5588636448e+00 | H | -1.4757464074e+01 | -2.0950246762e+00 | 6.5143169943e+00 |
| C | 1.1458313668e+01 | -7.2719272984e+00 | 4.9507834415e+00 | C | 1.2273404869e+01 | -7.9047920892e+00 | 5.3482373408e+00 |
| N | 1.2357320265e+01 | -7.8787769849e+00 | 5.3220079055e+00 | N | 1.1486892327e+01 | -7.2029474224e+00 | 4.8975721758e+00 |
| H | 1.0622066601e+01 | -6.7074558005e+00 | 4.6054822286e+00 | H | 1.3005004033e+01 | -8.5576418852e+00 | 5.7674269917e+00 |
| C | -3.5301184750e+00 | 1.3931429415e+01 | 3.0525985187e+00 | C | -3.6164328261e+00 | 1.3626519520e+01 | 3.2524367743e+00 |
| N | -4.0576550178e+00 | 1.4240428802e+01 | 4.0223982824e+00 | N | -3.9654820076e+00 | 1.4566039435e+01 | 3.8089922559e+00 |
| H | -3.0394215230e+00 | 1.3644011667e+01 | 2.1505022407e+00 | H | -3.2917422699e+00 | 1.2752596738e+01 | 2.7347446167e+00 |

| | | | | | | | |
|---|---|---|---|---|---|---|---|
| C | -6.4299567253e+00 | -1.2850729942e+01 | 1.8747416129e+00 | C | -6.7488517259e+00 | -1.3738453758e+01 | 1.8749008914e+00 |
| N | -7.3091562126e+00 | -1.3571172640e+01 | 2.0239313775e+00 | N | -6.9686126475e+00 | -1.2623181318e+01 | 2.0237616567e+00 |
| H | -5.6121466699e+00 | -1.2180579790e+01 | 1.7359769065e+00 | H | -6.5444219514e+00 | -1.4775857326e+01 | 1.7364362362e+00 |
| C | 1.3665320858e+01 | 5.5054058711e+00 | 3.7743035730e-01 | C | 1.4218486082e+01 | 4.6326160179e+00 | 5.7034661201e-01 |
| N | 1.4532654203e+01 | 4.7574958608e+00 | 4.2891327581e-01 | N | 1.3941936194e+01 | 5.6895397606e+00 | 2.2289968351e-01 |
| H | 1.2858551897e+01 | 6.2011074666e+00 | 3.2954205788e-01 | H | 1.4475716048e+01 | 3.6494839610e+00 | 8.9354045331e-01 |
| C | -1.3829924992e+01 | 5.9429688509e+00 | -1.6163608038e+00 | C | -1.3677106839e+01 | 5.9432805289e+00 | -6.3555430098e-01 |
| N | -1.3823592206e+01 | 5.4561029483e+00 | -5.7847425165e-01 | N | -1.3986783821e+01 | 5.4557702425e+00 | -1.6258678340e+00 |
| H | -1.3835803833e+01 | 6.3958380553e+00 | -2.5817856215e+00 | H | -1.3389060251e+01 | 6.3967470050e+00 | 2.8562292068e-01 |
| C | 5.9882750976e+00 | -1.3760523020e+01 | -2.9010733841e+00 | C | 5.7680590153e+00 | -1.3331626091e+01 | -2.8536348765e+00 |
| N | 6.5524518211e+00 | -1.2972841696e+01 | -2.2882522326e+00 | N | 6.7876183925e+00 | -1.3430854970e+01 | -2.3389123178e+00 |
| H | 5.4634851406e+00 | -1.4493200530e+01 | -3.4711166479e+00 | H | 4.8196803065e+00 | -1.3239336412e+01 | -3.3324151971e+00 |
| C | 4.7499748489e+00 | 1.3998047129e+01 | -3.7970253123e+00 | C | 4.2013926324e+00 | 1.4288970106e+01 | -3.8963805364e+00 |
| N | 3.8627782460e+00 | 1.3538542362e+01 | -4.3591859327e+00 | N | 4.4486028047e+00 | 1.3227869054e+01 | -4.2530858694e+00 |
| H | 5.5752309081e+00 | 1.4425477529e+01 | -3.2741239398e+00 | H | 3.9714541425e+00 | 1.5275990272e+01 | -3.5645829484e+00 |
| C | -1.1522504528e+01 | -6.9465325957e+00 | -5.5604671363e+00 | C | -1.2135611906e+01 | -6.9641159221e+00 | -4.9567066937e+00 |
| N | -1.2658893265e+01 | -7.0488199016e+00 | -5.4489101913e+00 | N | -1.2004162277e+01 | -7.0300411805e+00 | -6.0936595783e+00 |
| H | -1.0465458052e+01 | -6.8513741477e+00 | -5.6642350190e+00 | H | -1.2257886710e+01 | -6.9028059150e+00 | -3.8991339486e+00 |
| C | 1.2650700862e+01 | -3.2875900808e+00 | -6.9462379184e+00 | C | 1.2588119819e+01 | -2.8202263341e+00 | -7.2857323119e+00 |
| N | 1.3372666018e+01 | -2.3991973638e+00 | -6.8844989576e+00 | N | 1.3439495560e+01 | -2.8982894116e+00 | -6.5219571417e+00 |
| H | 1.1979132467e+01 | -4.1139483436e+00 | -7.0036667323e+00 | H | 1.1796177991e+01 | -2.7476188454e+00 | -7.9961733360e+00 |
| C | -6.9659044031e+00 | 9.7464666186e+00 | -8.4719585363e+00 | C | -6.6742409092e+00 | 1.0429183282e+01 | -8.3367991319e+00 |
| N | -7.4238927090e+00 | 1.0732524453e+01 | -8.1083328160e+00 | N | -7.7353571509e+00 | 1.0003459045e+01 | -8.2526670839e+00 |
| H | -6.5398876070e+00 | 8.8292518638e+00 | -8.8101847013e+00 | H | -5.6872059173e+00 | 1.0825172359e+01 | -8.4150597951e+00 |
| C | -1.4085463440e+00 | -1.1220040483e+01 | -9.1203126822e+00 | C | -1.1831976003e+00 | -1.1647381679e+01 | -1.0038957015e+01 |
| N | -1.5494252532e+00 | -1.1582638053e+01 | -1.0198720370e+01 | N | -1.7900738473e+00 | -1.1126286140e+01 | -9.2177093870e+00 |
| H | -1.2775122106e+00 | -1.0882767341e+01 | -8.1171940255e+00 | H | -6.1868570196e-01 | -1.2132084436e+01 | -1.0802867815e+01 |
| C | 8.3772518397e+00 | 6.7484787309e+00 | -1.0921636776e+01 | C | 8.1485704659e+00 | 6.1864063775e+00 | -1.0760942564e+01 |
| N | 7.2691031747e+00 | 6.4594590086e+00 | -1.0974183354e+01 | N | 7.5133102048e+00 | 7.0596908304e+00 | -1.1145787561e+01 |
| H | 9.4080316022e+00 | 7.0173196481e+00 | -1.0872771674e+01 | H | 8.7394684936e+00 | 5.3740853092e+00 | -1.0402970277e+01 |
| C | -9.1523346614e+00 | 7.0026831337e-01 | -1.2262679122e+01 | C | -8.1646635426e+00 | 3.8189164036e-01 | -1.2199232710e+01 |
| N | -8.2555092992e+00 | -2.2453321117e-03 | -1.2134359779e+01 | N | -9.3102331703e+00 | 3.3774486765e-01 | -1.2202113163e+01 |
| H | -9.9865493838e+00 | 1.3537297052e+00 | -1.2382028031e+01 | H | -7.0990765242e+00 | 4.2296786473e-01 | -1.2196548349e+01 |
| C | 4.8897833264e+00 | -5.1107373535e+00 | -1.2942054940e+01 | C | 5.1528960692e+00 | -5.0275878504e+00 | -1.3736074148e+01 |
| N | 4.7155009287e+00 | -4.4311883043e+00 | -1.3848766439e+01 | N | 4.4345251528e+00 | -4.5199821517e+00 | -1.3000841744e+01 |
| H | 5.0519015730e+00 | -5.7428311453e+00 | -1.2098642071e+01 | H | 5.8211209071e+00 | -5.4997514460e+00 | -1.4419965411e+01 |
| C | -2.6891184046e-01 | 4.5746347974e+00 | -1.4688153866e+01 | C | -6.0845314225e-01 | 3.8365071744e+00 | -1.4171294886e+01 |
| N | -1.0395957521e-01 | 3.5732807782e+00 | -1.4154892312e+01 | N | 2.5863414226e-01 | 4.3615188156e+00 | -1.4706840798e+01 |
| H | -4.2233788139e-01 | 5.5060728673e+00 | -1.5184191896e+01 | H | -1.4150097206e+00 | 3.3481596075e+00 | -1.3673145013e+01 |
| C | 1.5330559028e+00 | 1.4918063717e+00 | -1.4596662148e+01 | C | 1.8294294465e+00 | 2.4280199306e+00 | -1.5085571758e+01 |
| N | 1.8139029701e+00 | 2.5424128436e+00 | -1.4959481624e+01 | N | 1.4974098462e+00 | 1.5426393492e+00 | -1.4437381032e+01 |
| H | 1.2718274496e+00 | 5.1454706379e-01 | -1.4259180155e+01 | H | 2.1382613482e+00 | 3.2515923817e+00 | -1.5688499142e+01 |

**HCN_CONFIG_03**

| | | | |
|---|---|---|---|
| C | 5.3799706591e-01 | 4.2070529038e-02 | 3.1270467434e+00 |
| N | -5.7452131824e-01 | -4.4927481956e-02 | 2.8643278873e+00 |
| H | 1.5728394093e+00 | 1.2300693752e-01 | 3.3714250931e+00 |
| C | -2.1895356090e+00 | 2.5591529798e+00 | -1.5196045456e-01 |
| N | -2.2362091595e+00 | 1.4576251867e+00 | 1.6227688756e-01 |
| H | -2.1461334880e+00 | 3.5837741459e+00 | -4.4425803836e-01 |
| C | 6.0672520865e-02 | -5.1403264159e-01 | -3.1985974218e+00 |
| N | -6.4791526496e-02 | 5.4893038278e-01 | -2.7879206779e+00 |
| H | 1.7737777687e-01 | -1.5027854924e+00 | -3.5805893576e+00 |
| C | -1.9246763085e-01 | -3.2217037784e-02 | -5.4810877522e+00 |
| N | 2.0553338821e-01 | 3.4404396214e-02 | -6.5541412363e+00 |
| H | -5.6266921941e-01 | -9.4190717610e-02 | -4.4829493838e+00 |
| C | 4.3449432528e+00 | -3.4544831853e+00 | -3.2015728665e+00 |
| N | 3.2831600728e+00 | -3.5691841769e+00 | -2.7847429641e+00 |
| H | 5.3325957001e+00 | -3.3477798357e+00 | -3.5892933770e+00 |
| C | -5.9800572742e-01 | 6.5229235305e+00 | -6.2870471415e-02 |
| N | -4.4611706896e-01 | 5.3940682152e+00 | 6.7139216830e-02 |
| H | -7.3927874715e-01 | 7.5729644040e+00 | -1.8380951993e-01 |
| C | -3.4887532777e+00 | -4.5559517454e+00 | 3.1158305180e+00 |
| N | -2.8121150262e+00 | -3.6620413266e+00 | 2.8763063099e+00 |
| H | -4.1181433233e+00 | -5.3874575565e+00 | 3.3386228255e+00 |
| C | 3.3374733127e-01 | -4.4110532230e-01 | 6.0372888839e+00 |
| N | -3.5640522515e-01 | 4.7105186855e-01 | 5.9601804253e+00 |
| H | 9.7571247926e-01 | -1.2895793795e+00 | 6.1090013569e+00 |
| C | 3.0756881957e-01 | -2.1553122756e-01 | 9.1488597813e+00 |
| N | 1.2936062563e+00 | 2.3016430838e-01 | 8.7702139455e+00 |
| H | -6.0962152421e-01 | -6.3012024019e-01 | 9.5010642384e+00 |
| C | -5.3508333044e+00 | 4.4713258552e+00 | 6.2904233773e+00 |
| N | -4.7079013909e+00 | 4.7725229522e+00 | 5.3903090125e+00 |
| H | -5.9488702884e+00 | 4.1911663164e+00 | 7.1277002457e+00 |
| C | 4.8548335390e-01 | -7.9255218733e+00 | 3.2536518648e+00 |
| N | 1.0068183522e+00 | -8.9159879431e+00 | 3.0057127785e+00 |
| H | 5.5784118133e-04 | -7.0042024528e+00 | 3.4842769464e+00 |
| C | 5.9676795860e+00 | 7.2769940661e+00 | 3.1538757951e-01 |
| N | 4.9330345850e+00 | 6.9754625035e+00 | 7.0638047589e-01 |
| H | 6.9300831746e+00 | 7.5574708994e+00 | -4.8318668005e-02 |
| C | -8.2702338459e+00 | -1.2321722498e+00 | -1.7520821737e+00 |
| N | -9.0096398218e+00 | -1.8400579990e+00 | -2.3829868615e+00 |
| H | -7.5824601762e+00 | -6.6671831190e-01 | -1.1652266726e+00 |
| C | 6.4982971409e+00 | -4.7043212907e+00 | -4.6786898604e+00 |
| N | 6.6079020013e+00 | -3.5940299045e+00 | -4.4150104746e+00 |
| H | 6.3963570773e+00 | -5.7370954686e+00 | -4.9239568105e+00 |
| C | -1.3813869295e+00 | 5.1959445531e+00 | -7.3922515282e+00 |
| N | -1.5925282075e+00 | 5.8629821424e+00 | -6.4840845713e+00 |
| H | -1.1849757618e+00 | 4.5754758007e+00 | -8.2370067213e+00 |
| C | 2.9062381801e-01 | 2.5069982884e-01 | -9.2881301217e+00 |
| N | 4.3726222545e-01 | 1.1717711214e+00 | -8.6214878954e+00 |
| H | 1.5423267149e-01 | -6.0605929202e-01 | -9.9082360962e+00 |
| C | -1.0429088223e+00 | 5.6954234238e-02 | -1.2505788189e+01 |
| N | -1.0490308055e+00 | -6.0820866024e-02 | -1.1365446769e+01 |
| H | -1.0372016441e+00 | 1.6650826156e-01 | -1.3566512233e+01 |
| C | 5.0507718376e+00 | -5.2973984127e+00 | -9.5616475512e+00 |
| N | 6.0111550616e+00 | -4.7907142386e+00 | -9.1939218148e+00 |
| H | 4.1574481592e+00 | -5.7687138448e+00 | -9.9037070852e+00 |
| C | -1.1489367132e+00 | 9.2032518122e+00 | -7.4672465092e+00 |

| | | | |
|---|---|---|---|
| N | -5.0996798023e-01 | 9.9630853953e+00 | -6.8939587187e+00 |
| H | -1.7432913195e+00 | 8.4964601094e+00 | -8.0004990258e+00 |
| C | -6.7275490320e+00 | -8.8753437352e+00 | -4.5861878736e+00 |
| N | -6.4993487470e+00 | -8.3699824708e+00 | -5.5895928909e+00 |
| H | -6.9398063612e+00 | -9.3454158545e+00 | -3.6528341440e+00 |
| C | 1.1188714059e+01 | 2.4804163241e+00 | -3.1856298429e+00 |
| N | 1.1711780677e+01 | 1.5363267168e+00 | -2.7991286998e+00 |
| H | 1.0702167466e+01 | 3.3585861195e+00 | -3.5451578366e+00 |
| C | -1.0126732059e+01 | 6.5620014734e+00 | -4.2633093100e-01 |
| N | -1.0056012830e+01 | 6.2684491741e+00 | -1.5322752536e+00 |
| H | -1.0192520478e+01 | 6.8350477581e+00 | 6.0240021270e-01 |
| C | 3.2814489502e+00 | -1.2037305902e+01 | 8.6483121118e-01 |
| N | 2.9132579952e+00 | -1.1018201877e+01 | 1.2391965585e+00 |
| H | 3.6239279575e+00 | -1.2985263089e+01 | 5.1661366786e-01 |
| C | 5.1045396963e+00 | 1.0735407026e+01 | 3.2712652910e+00 |
| N | 5.6171523579e+00 | 9.8469889556e+00 | 2.7591826833e+00 |
| H | 4.6277280464e+00 | 1.1561800202e+01 | 3.7476003888e+00 |
| C | -1.0635066081e+01 | -4.1045766852e+00 | 5.1659231760e+00 |
| N | -9.8580199787e+00 | -3.3644848124e+00 | 4.7624998477e+00 |
| H | -1.1357868004e+01 | -4.7929927648e+00 | 5.5411708759e+00 |
| C | 9.0759172874e+00 | -3.2080832269e+00 | 6.9964385236e+00 |
| N | 9.0970863352e+00 | -4.3300154059e+00 | 6.7616895862e+00 |
| H | 9.0562366031e+00 | -2.1644811485e+00 | 7.2147903690e+00 |
| C | -3.6610919946e+00 | 7.4466665879e+00 | 9.2733845506e+00 |
| N | -3.2887701398e+00 | 7.4303727036e+00 | 8.1892268351e+00 |
| H | -4.0074277330e+00 | 7.4618141945e+00 | 1.0281844893e+01 |
| C | -1.2652379246e+00 | -5.6748040715e+00 | 1.0289270639e+01 |
| N | -2.1990749660e+00 | -5.2585618105e+00 | 1.0807901304e+01 |
| H | -3.9659298801e-01 | -6.0619760144e+00 | 9.8068558625e+00 |
| C | -1.1320543200e+00 | -9.6031306194e-01 | 1.2210728631e+01 |
| N | -6.6231981079e-01 | -5.8906585692e-02 | 1.1680537287e+01 |
| H | -1.5689817390e+00 | -1.7987864056e+00 | 1.2703909164e+01 |
| C | 2.8062332734e+00 | 5.0994542190e-01 | 1.4853974641e+01 |
| N | 2.3895311413e+00 | -5.4456531131e-01 | 1.4684701332e+01 |
| H | 3.1938496471e+00 | 1.4908301260e+00 | 1.5011419551e+01 |
| C | -6.4822845246e+00 | 6.3919131728e+00 | 1.2470542647e+01 |
| N | -6.4703373358e+00 | 5.4429549877e+00 | 1.1827407642e+01 |
| H | -6.4934102846e+00 | 7.2746156240e+00 | 1.3068775674e+01 |
| C | 1.2942909187e+00 | -1.1118985791e+01 | 9.8702957374e+00 |
| N | 5.9692194851e-01 | -1.0676760204e+01 | 1.0665531587e+01 |
| H | 1.9429615648e+00 | -1.1530333900e+01 | 9.1305733016e+00 |
| C | 6.9914741071e+00 | 9.8321821649e+00 | 8.3414877739e+00 |
| N | 8.1010245210e+00 | 9.8049013385e+00 | 8.6286125585e+00 |
| H | 5.9593878164e+00 | 9.8575567544e+00 | 8.0744219125e+00 |
| C | -1.3389163939e+01 | -1.8753858925e+00 | 6.5457301777e+00 |
| N | -1.2948332906e+01 | -2.8168134981e+00 | 7.0291234649e+00 |
| H | -1.3799224584e+01 | -9.9969476097e-01 | 6.0960797970e+00 |
| C | 1.1467608607e+01 | -7.4177712320e+00 | 4.8069575246e+00 |
| N | 1.2347393685e+01 | -7.7230315954e+00 | 5.4755989328e+00 |
| H | 1.0649251117e+01 | -7.1338338545e+00 | 4.1849923005e+00 |
| C | -4.2708618211e+00 | 1.4072070648e+01 | 3.2543080840e+00 |
| N | -3.2666230770e+00 | 1.4090240558e+01 | 3.8069937139e+00 |
| H | -5.2049904867e+00 | 1.4055160983e+01 | 2.7402187385e+00 |
| C | -6.4064290869e+00 | -1.2881682312e+01 | 2.0193750201e+00 |
| N | -7.3342809608e+00 | -1.3538119139e+01 | 1.8694797305e+00 |
| H | -5.5433650128e+00 | -1.2271066941e+01 | 2.1588003711e+00 |

| | | | |
|---|---|---|---|
| C | 1.4121159886e+01 | 4.6184891241e+00 | 2.2868952751e-01 |
| N | 1.4045869870e+01 | 5.7046256531e+00 | 5.8775200149e-01 |
| H | 1.4191180575e+01 | 3.6081840463e+00 | -1.0530285058e-01 |
| C | -1.3963383543e+01 | 6.2270034148e+00 | -1.2517811889e+00 |
| N | -1.3681073213e+01 | 5.1527847782e+00 | -9.6780547766e-01 |
| H | -1.4225972748e+01 | 7.2262258210e+00 | -1.5159244233e+00 |
| C | 6.2565146157e+00 | -1.2843041002e+01 | -2.7440860646e+00 |
| N | 6.2660016793e+00 | -1.3952610771e+01 | -2.4558987481e+00 |
| H | 6.2476859901e+00 | -1.1810936897e+01 | -3.0121407727e+00 |
| C | 3.9397767468e+00 | 1.3465552400e+01 | -3.8123841242e+00 |
| N | 4.7279798979e+00 | 1.4107188569e+01 | -4.3427856945e+00 |
| H | 3.2066132271e+00 | 1.2868711872e+01 | -3.3190051685e+00 |
| C | -1.2447601698e+01 | -7.0146914086e+00 | -5.0991388671e+00 |
| N | -1.1670991245e+01 | -6.9760335025e+00 | -5.9415572765e+00 |
| H | -1.3169997221e+01 | -7.0506414517e+00 | -4.3155430148e+00 |
| C | 1.2503653331e+01 | -2.6107961345e+00 | -6.9086195303e+00 |
| N | 1.3529695563e+01 | -3.1219386733e+00 | -6.9246709611e+00 |
| H | 1.1549253225e+01 | -2.1353302492e+00 | -6.8936935116e+00 |
| C | -7.5222569595e+00 | 9.8061380674e+00 | -8.4416398526e+00 |
| N | -6.8297698354e+00 | 1.0668802275e+01 | -8.1407095376e+00 |
| H | -8.1663883089e+00 | 9.0036957921e+00 | -8.7215529732e+00 |
| C | -1.6062978016e+00 | -1.0877494264e+01 | -9.4923623984e+00 |
| N | -1.3382495945e+00 | -1.1948440483e+01 | -9.8014115229e+00 |
| H | -1.8556222284e+00 | -9.8813155708e+00 | -9.2048984942e+00 |
| C | 8.3846002059e+00 | 6.5812346223e+00 | -1.0839831732e+01 |
| N | 7.2612560336e+00 | 6.6380567424e+00 | -1.1061543535e+01 |
| H | 9.4295128962e+00 | 6.5283895930e+00 | -1.0633591512e+01 |
| C | -8.8512118079e+00 | 9.9186958215e-02 | -1.1730019252e+01 |
| N | -8.5770763128e+00 | 6.3964310693e-01 | -1.2703181051e+01 |
| H | -9.1061952762e+00 | -4.0353837511e-01 | -1.0824800590e+01 |
| C | 4.3471215693e+00 | -4.4891057911e+00 | -1.3273839178e+01 |
| N | 5.2950035380e+00 | -5.0950221660e+00 | -1.3494456780e+01 |
| H | 3.4654256571e+00 | -3.9254829285e+00 | -1.3068627719e+01 |
| C | -7.0069681218e-01 | 4.2680412075e+00 | -1.4311506771e+01 |
| N | 3.5713996726e-01 | 3.9006893196e+00 | -1.4557109998e+01 |
| H | -1.6846817507e+00 | 4.6097355844e+00 | -1.4083058201e+01 |
| C | 1.6075571187e+00 | 2.5416312792e+00 | -1.4872516378e+01 |
| N | 1.7343440365e+00 | 1.4213153392e+00 | -1.4664900679e+01 |
| H | 1.4896301824e+00 | 3.5837309362e+00 | -1.5065626964e+01 |

# References


(S1) Miao, Y.; Feher, V. A.; McCammon, J. A. Gaussian Accelerated Molecular Dynamics: Unconstrained Enhanced Sampling and Free Energy Calculation. *J. Chem. Theory Comput.* **2015**, *11*, 3584–3595.

(S2) Zhao, Y.; Zhang, J.; Zhang, H.; Gu, S.; Deng, Y.; Tu, Y.; Hou, T.; Kang, Y. Sig-

moid Accelerated Molecular Dynamics: An Efficient Enhanced Sampling Method for Biosystems. *J. Phys. Chem. Lett.* **2023**, *14*, 1103–1112.

(S3) Stan-Bernhardt, A.; Glinkina, L. Processing of Computational Nanoreactor Simulations. `https://github.com/ochsenfeld-lab/nanoreactor_processing`, accessed 2023-10-01.

(S4) Landrum, G. rdkit/rdkit: 2021_03_4 (Q1 2021) Release. 2021; `https://zenodo.org/record/5085999`, accessed 2023-10-01.

(S5) Haslwanter, T. *An Introduction to Statistics with Python*, 1st ed.; Springer: Cham, 2016.

(S6) Benner, S. A.; Bell, E. A.; Biondi, E.; Brasser, R.; Carell, T.; Kim, H.; Mojzsis, S. J.; Omran, A.; Pasek, M. A.; Trail, D. When Did Life Likely Emerge on Earth in an RNA-First Process? *ChemSystemsChem* **2020**, *2*, e1900035.

(S7) Marks, J. H.; Wang, J.; Kleimeier, N. F.; Turner, A. M.; Eckhardt, A. K.; Kaiser, R. I. Prebiotic Synthesis and Isomerization in Interstellar Analog Ice: Glycinal, Acetamide, and Their Enol Tautomers. *Angew. Chem. Int. Ed.* **2023**, *62*, e202218645.

(S8) Teichert, J. S.; Kruse, F. M.; Trapp, O. Direct Prebiotic Pathway to DNA Nucleosides. *Angew. Chem., Int. Ed.* **2019**, *58*, 9944–9947.
